# Characterization of Potential Threats from Cyanobacterial Toxins in Lake Victoria Embayments and during Water Treatment

**DOI:** 10.3390/toxins14100664

**Published:** 2022-09-23

**Authors:** Mark Olokotum, Jean-François Humbert, Catherine Quiblier, William Okello, Ronald Semyalo, Marc Troussellier, Benjamin Marie, Kathrin Baumann, Rainer Kurmayer, Cécile Bernard

**Affiliations:** 1 Department of Zoology, Entomology and Fisheries Sciences, Makerere University, Kampala 7062, Uganda; 2 National Fisheries Resources Research Institute (NaFIRRI), Jinja 343, Uganda; 3 Research Department for Limnology, University of Innsbruck, 5310 Mondsee, Austria; 4 Institute of Ecology and Environmental Sciences of Paris, INRAE-Sorbonne University, 75005 Paris, France; 5 UMR Molecules of Communication and Adaptations of Microorganisms (MCAM) CNRS-MNHN, Muséum National d’Histoire Naturelle, 75005 Paris, France; 6 Department of Ecology, Campus Grands Moulins, Université de Paris, 75013 Paris, France; 7 UMR Marine Biodiversity Exploitation and Conservation (MARBEC), CNRS-Université de Montpellier-IFREMER-IRD, CEDEX 5, 34095 Montpellier, France

**Keywords:** drinking water, rapid sand filtration, recreational areas, exposure routes, *Microcystis*, *Dolichospermum*, microcystins

## Abstract

Africa’s water needs are often supported by eutrophic water bodies dominated by cyanobacteria posing health threats to riparian populations from cyanotoxins, and Lake Victoria is no exception. In two embayments of the lake (Murchison Bay and Napoleon Gulf), cyanobacterial surveys were conducted to characterize the dynamics of cyanotoxins in lake water and water treatment plants. Forty-six cyanobacterial taxa were recorded, and out of these, fourteen were considered potentially toxigenic (i.e., from the genera *Dolichospermum*, *Microcystis*, *Oscillatoria*, *Pseudanabaena* and *Raphidiopsis*). A higher concentration (ranging from 5 to 10 µg MC-LR equiv. L^−1^) of microcystins (MC) was detected in Murchison Bay compared to Napoleon Gulf, with a declining gradient from the inshore (max. 15 µg MC-LR equiv. L^−1^) to the open lake. In Murchison Bay, an increase in *Microcystis* sp. biovolume and MC was observed over the last two decades. Despite high cell densities of toxigenic *Microcystis* and high MC concentrations, the water treatment plant in Murchison Bay efficiently removed the cyanobacterial biomass, intracellular and dissolved MC to below the lifetime guideline value for exposure via drinking water (<1.0 µg MC-LR equiv. L^−1^). Thus, the potential health threats stem from the consumption of untreated water and recreational activities along the shores of the lake embayments. MC concentrations were predicted from *Microcystis* cell numbers regulated by environmental factors, such as solar radiation, wind speed in the N–S direction and turbidity. Thus, an early warning through microscopical counting of *Microcystis* cell numbers is proposed to better manage health risks from toxigenic cyanobacteria in Lake Victoria.

## 1. Introduction

For decades, the increase in cyanobacterial dominance and regime shifts in freshwater ecosystems has been linked to a multitude of increasing human activities in the catchment, resulting in greater nutrient pollution pressures [1]. Moreover, this dominance has been predicted to increase globally because of climate change [2]. The changes will be system specific, depending on the mean depth of the mixed water layer, the light penetration and light absorption characteristics specific to the cyanobacterial taxa [3]. The cyanobacterial blooms have attracted attention due to their impact on the aquatic ecosystem services, including water supply, fish food, recreational activities [4], not least because of the health threats associated with cyanotoxins [5].

Lake Victoria, the second largest freshwater lake in the world, is a major source of water for the riparian populations and supports one of the largest freshwater fisheries in the world (1 million tons of fish annually), but increasing pollution has threatened it through widespread eutrophication [6]. After the occurrence of fish kills in Nyanza Gulf that were attributed to suspended algae and detritus coupled with low dissolved oxygen [7], several studies have focused on phytoplankton composition, cyanobacteria and cyanotoxins in the open lake and the embayments (for review, see Ref [8]). Most of the studies on cyanotoxins in Lake Victoria have detected microcystins (MC) produced by dominant cyanobacteria, most importantly, *Microcystis* spp., and possibly *Dolichospermum* spp. An earlier study by Kotut et al. [9] conducted during a *Dolichospermum* bloom examined water samples for potential anatoxin-a (ATX), which was undetectable in both environmental samples and isolated strains. More recently, very low concentrations of cylindrospermopsin (CYN) and nodularin (NOD) have been reported in the southern part of Lake Victoria [10].

Since the first report of MC occurrence in Nyanza Gulf (Kenya) at a concentration <1 µg MC-LR equiv. L^−1^ [11], several studies have reported higher MC concentrations across the embayments of Lake Victoria. For instance, from the inner part of Nyanza Gulf, a maximum of 274 (133) µg MC-LR equiv. L^−1^ has been reported from surface samples dominated by *Microcystis* spp. In patches formed in the open water, even up to 2 mg MC-LR equiv. L^−1^ has been reported, probably through concentrating buoyant *Microcystis* colonies along the Langmuir spirals [12]. In general, high spatial variability has been observed, and MC concentrations did not exceed 5 µg MC-LR equiv. L^−1^ in most of the embayments, i.e., <2 µg MC-LR equiv. L^−1^ in the Napoleon Gulf and Murchison Bay [8,13] and <4 µg MC-LR equiv. L^−1^ in closed bays in Tanzania [8,14]. Therefore, these studies have shown that the Lake Victoria riparian populations (lake shore residents, fishermen, recreationists and all people connected to treated water) are potentially exposed to variable concentrations of toxigenic cyanobacteria and MC.

The embayments of Lake Victoria serve as the source of water and abstraction for most water treatment facilities [8]. Therefore, the location and technology of the water intake, as well as the efficiency of clarification through flocculation, rapid sand filtration and chlorination treatment, are important factors in the water treatment process [4]. The major exposure routes for cyanotoxins to lakeside populations include drinking and other domestic use (both treated and untreated water), eating contaminated fish and food supplements, and recreational activities [4]. Therefore, lakeside residents, fishermen and recreationists are potentially exposed to direct consumption of toxigenic cyanobacteria and intracellular toxins, while those connected to treated water could be exposed to dissolved cyanotoxins. The dangers of cyanotoxins originate from both cell-bound and free dissolved toxins [4,15]. Cyanotoxins, such as MC, NOD, CYN and ATX, which are cell bound, can be released into the water upon cyanobacterial cell lysis [16]. For example, up to 90% of the CYNs were found dissolved in water in temperate lakes dominated by *Aphanizomenon* sp. [17]. Thus, it is generally accepted that dissolved cyanotoxins are the main threat to humans through the consumption of untreated water for domestic use or accidental consumption of water during recreation [5]. These exposure routes have been largely studied in the northern hemisphere of the globe where national regulations to prevent health risks from cyanotoxins have been established, which contrasts with the south [18]. Currently, the populations in the east African countries with access to Lake Victoria lack community awareness of the dangers of exposure to cyanotoxins and have no regular monitoring or regulations mitigating the effects of cyanotoxins from treated or untreated water.

In the present work, we characterized the conditions and relative risks associated with toxigenic cyanobacteria and their toxins for human populations for a one-year period (November 2017–October 2018) from lake water (raw water) at two embayments of Lake Victoria in Uganda, Murchison Bay (MB) and Napoleon Gulf (NG), and during water treatment (November 2016–January 2017). The two embayments differ in eutrophication status, with higher nutrient concentrations in MB than in NG, and show contrasting abundances of the dominant toxigenic cyanobacteria: *Microcystis aeruginosa* and *M. flos-aquae* in MB and *Dolichospermum circinale* and *Planktolyngbya circumcreta* in NG [19]. From the point of view of sanitation, no studies have been conducted on both the source of cyanotoxin exposure (dynamics in lake water and removal during water treatment) and the associated health hazards. We monitored the dynamics and the spatial variability of toxigenic cyanobacteria and cyanotoxins in lake water from the inshore to the abstraction point of the water treatment plants (WTPs), as well as in the open lake. We also determined the removal of MC after water treatment in two WTPs, one in MB and one in NG, supplying treated water for the cities Kampala and Jinja, respectively. In considering extracellular MC, we also measured the intracellular MC level and the potential cyanobacterial cell lysis during the water treatment process. Afterward, the potential risks associated with the toxigenic cyanobacteria and their toxins for human populations using raw and treated water from these two embayments were discussed.

## 2. Results

**Figure 1:**
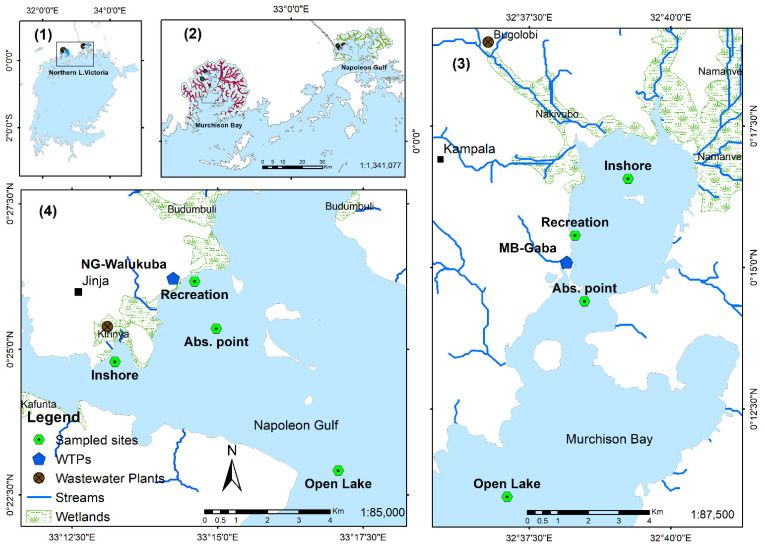
Location of the two study areas in Murchison Bay (MB) and Napoleon Gulf (NG) (**1**) and close up with tributaries and wetlands (**2**) and the location of sampling sites (Inshore, Recreation, WTP abstraction points (Abs. point) and open lake) in MB (**3**) and NG (**4**). The sampling depths in MB were as follows: Inshore = 1.4–2.5 m (1.84 ± 0.34), Recreation = 2–2.7 m (2.3 ± 0.3), WTP abstraction points (Abs. point) = 9.4–18.4 m (14 ± 2.33) and Open Lake = 12–14 m (12.7 ± 0.5). In NG, the sampling depths were as follows: Inshore = 5.4–6.5 m (6.1 ± 0.3), Recreation = 5.7–7.2 m (6.5 ± 0.5), WTP abstraction points (Abs. point) = 11.4–15.8 m (13.1 ± 1.2) and Open Lake = 16.7–21.5 m (18.1 ± 1.3).

To investigate the dynamics of cyanobacteria and cyanotoxins concentrations within the two embayments of MB and NG, the temporal–spatial variability was monitored at four stations: (i) the inshore station, (ii) the recreational area, (iii) in the lake water at the abstraction point of the WTP and (iv) in the open lake (Figure 1).

### 2.1. Toxigenic vs. Non-Toxigenic Cyanobacteria Diversity and Biovolume

**Figure 2:**
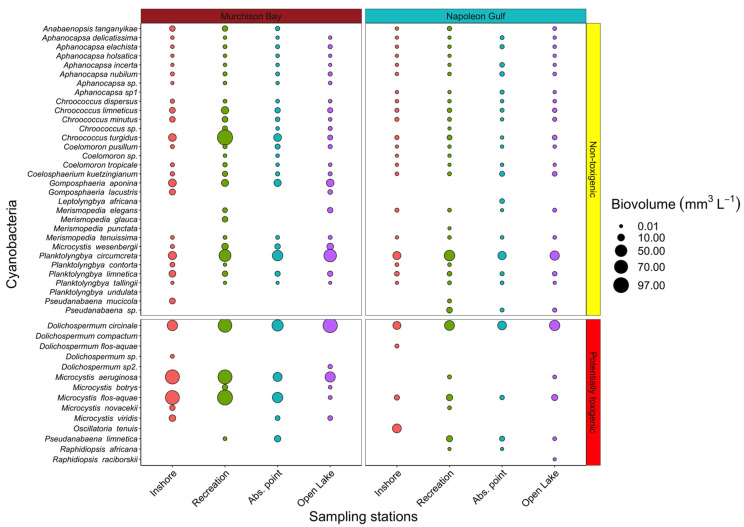
Potentially toxigenic and non-toxigenic cyanobacteria recorded from the lake survey at sampling sites: (i) inshore, (ii) recreational area, (iii) WTP abstraction point (Abs. point), (iv) open lake, in Murchison Bay and Napoleon Gulf. Among the 46 cyanobacteria species, 14 were considered potentially toxigenic. Circle sizes are proportional to the total biovolume (mm^3^ L^−1^) of each taxon (data collected between November 2017 and October 2018, *n* = 120).

*In the embayments*: From the lake survey, up to 46 cyanobacteria taxa were recorded with 14 potentially toxigenic taxa belonging to five genera: *Dolichospermum, Microcystis, Oscillatoria, Pseudanabaena* and *Raphidiopsis* (previously *Cylindrospermopsis*). Most of the cyanobacterial species identified so far were considered non-toxigenic (27 taxa from MB and 26 from NG; Figure 2). Comparing the two embayments, a higher cyanobacterial biovolume was observed in MB (0.9 × 10^−4^ to 45.0 mm^3^ L^−1^; mean ± SD = 2 ± 4.2 mm^3^ L^−1^) than NG (0.4 × 10^−4^ to 22 mm^3^ L^−1^; mean ± SD = 0.5 ± 1.4 mm^3^ L^−1^; Mann–Whitney test, *p* < 0.0001). In MB, higher cyanobacterial biovolumes were recorded from inshore samplings than at the WTP abstraction point and open lake stations (Friedman test, X^2^ = 4987.8, *p* < 0.001 with the Nemenyi post hoc test). However, in NG, no significant differences in the cyanobacteria biovolume were observed from the inshore to the open lake stations (repeated measures ANOVA, F = 0.618, *p* = 0.603; Figure 2).

In MB, the most dominant toxigenic cyanobacteria belonged to the genus *Microcystis* with *M. aeruginosa* (0.5–31.4 mm^3^ L^−1^, mean ± SD = 7.3 ± 6.7 mm^3^ L^−1^; 18.7% of the total cyanobacteria biovolume) and *M. flos-aquae* (0.4–30 mm^3^ L^−1^, mean ± SD = 6.6 ± 6.7 mm^3^ L^−1^; 17.9% of the total cyanobacteria biovolume). In MB, the highest biovolume of toxigenic cyanobacteria was recorded from the inshore and recreational stations (0.6–28.2 and 1.2–29.8 mm^3^ L^−1^, respectively). The non-toxigenic cyanobacteria made the most significant contribution to the cyanobacterial biovolume (56.4%), ranging from 8.9 × 10^−5^ to 45 mm^3^ L^−1^, mean ± SD = 1 ± 3 mm^3^ L^−1^, dominated by *Chroococcus turgidus* in the recreational area (Figure 2). The temporal dynamics of the most abundant toxigenic cyanobacteria in MB were dominated by *Microcystis* spp. at the inshore and recreational sites (with the highest biovolume observed in September (inshore station) and November (recreational station) (Figure S1)).

In NG, the dominant potentially toxigenic cyanobacterium was *D. circinale,* ranging from 0.1 to 8.6 mm^3^ L^−1^, mean ± SD = 2.3 ± 1.9 mm^3^ L^−1^ (36% of the total cyanobacteria biovolume), followed by *M. flos-aquae* with 0.3–2.9 mm^3^ L^−1^, 0.7 ± 0.6 mm^3^ L^−1^ (6.1% of the total cyanobacteria biovolume). The toxigenic cyanobacterium *Oscillatoria tenuis* (22 mm^3^ L^−1^) was detected once with a high abundance in November 2017 (62.3% of the total cyanobacteria biovolume) at the inshore station. The non-toxigenic cyanobacterial biovolume ranged from 0.4 × 10^−4^ to 5.5; 0.3 ± 0.7 mm^3^ L^−1^ (47% of the total cyanobacteria biovolume), dominated by *Planktolyngbya circumcreta* (0.5 to 5.5; 2 ± 1.3 mm^3^ L^−1^; 72% of the non-toxigenic cyanobacteria biovolume) in the recreational station (Figure 2 and Figure S1).

**Figure 3:**
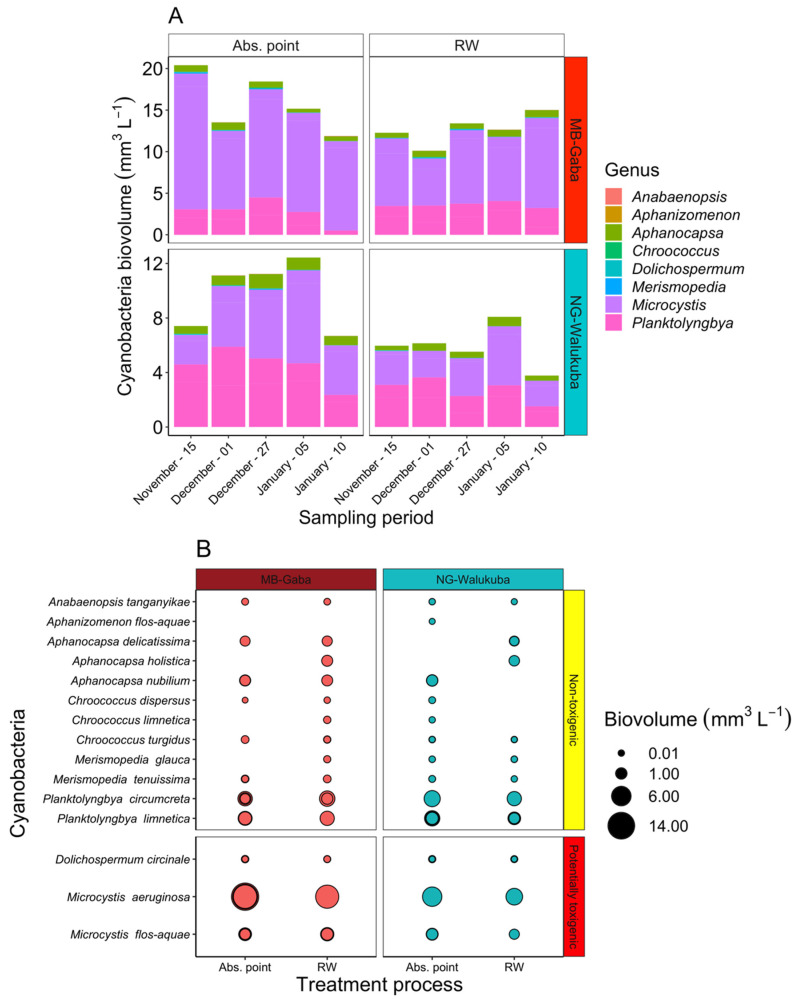
Cyanobacterial biovolume and composition at the WTP Abs. point and in raw water (RW) from MB-Gaba and NG-Walukuba water treatment plants. (**A**) Temporal dynamics of cyanobacteria genera; (**B**) Proportion of potentially toxigenic and non-toxigenic cyanobacteria (data collected between November 2016 and January 2017, *n* = 90).

*During water treatment:* Since our survey on MC concentrations during water treatment (see below) was performed for three months (November 2016–January 2017) only, the seasonal variation in cyanobacteria biovolume in raw water could not be considered. Thus, it became important to investigate the seasonality of cyanobacteria community composition for a longer period. When comparing the toxigenic cyanobacteria biovolume composition at WTP abstraction points for both study periods, on average higher (toxigenic) cyanobacteria biovolume was recorded (*t*-test, *p* = 0.04; Figure S2). During the water treatment survey, 15 cyanobacterial taxa were found in the abstracted water (and raw water entering the treatment process), including 3 potentially toxigenic taxa (*M. aeruginosa, M. flos-aquae* and *Dolichospermum* sp.) and 12 non-toxigenic cyanobacteria (Figure 3). The taxonomic composition recorded during the two study periods was similar (see Figure 2 and Figure 3). The depth-integrated water at the WTP abstraction point of MB-Gaba was dominated by cyanobacteria (15.9 ± 3.5 mm^3^ L^−1^; >90% of total phytoplankton biovolume), i.e. *Microcystis* spp. (12.3 ± 2.6 mm^3^ L^−1^), >70% of the total cyanobacterial biovolume), (Figure 3A). At the NG-Walukuba abstraction point, cyanobacteria were dominated by the non-toxigenic *Planktolyngbya* (4.5 ± 1.3 mm^3^ L^−1^; 46% of the total cyanobacterial biovolume) and the toxigenic *Microcystis* spp. (4.4 ± 1.7 mm^3^ L^−1^; 44% of the total cyanobacterial biovolume), followed by the non-toxigenic *Aphanocapsa* (0.8 ± 0.2 mm^3^ L^−1^; 8% of the total cyanobacterial biovolume). Other genera, such as *Dolichospermum* sp., *Chroococcus* sp. and *Merismopedia* sp., contributed < 1% of the cyanobacterial biovolume (Figure 3).

### 2.2. Cyanotoxins Occurrence in the Lake Victoria Embayments and during Water Treatment

**Figure 4:**
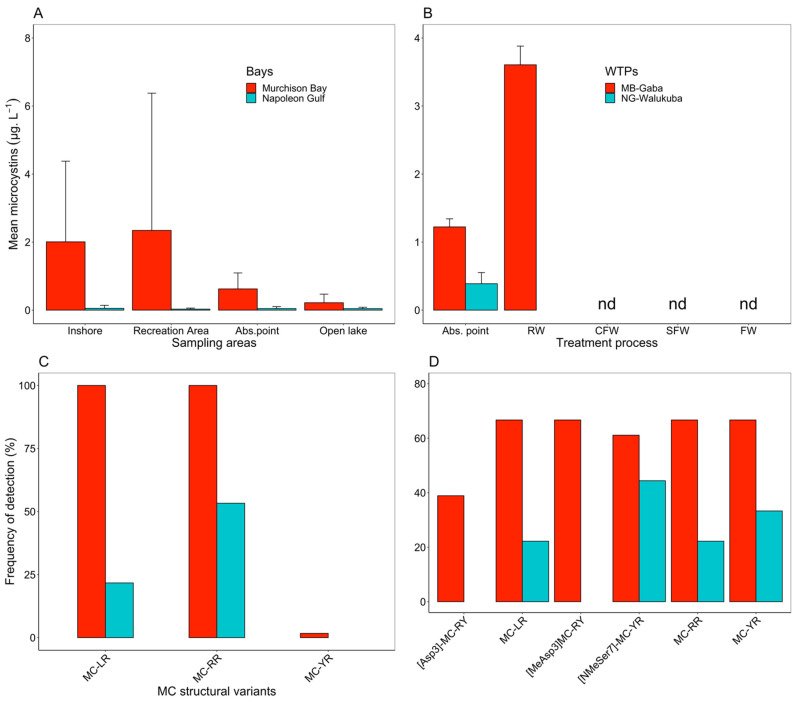
Spatial variation in the intracellular MC concentrations (mean ± SD), (**A**) at (i) inshore stations in the bays, (ii) recreational areas, (iii) WTP abstraction points (Abs. point) and (iv) open lake in Murchison Bay (MB) and Napoleon Gulf (NG), (between November 2017 and October 2018, *n* = 120), (**B**) during the water treatment from lake water to final water in MB-Gaba and NG-Walukuba (between November 2016 and January 2017, *n* = 90). Note the different scales in the *y*-axis. (**C**) Relative frequency of the structural variants of MC detected during the lake survey and (**D**) from the WTP abstraction points and raw water entering the WTPs (*n* = 18), from MB-Gaba and NG-Walukuba. Abbreviations: Abs. point = WTP abstraction point, RW = Raw water, CFW = Coagulated and flocculated water, SFW = Sand filtered water, FW = Final water, nd = not detected.

*In the embayments*: During the field survey, there was no detection of ATX, CYN or STX in all environmental samples (*n* = 120), but there was a detection of homoanatoxin-a (HTX) at the inshore station in MB (at low HTX concentrations, <0.04 HTX L^−1^) in 2 samples, while 99 samples (82.5%, *n* = 120) were found to contain MC. The most frequent MC congeners in MB and NG included MC-RR and MC-LR (Figure 4). The total concentrations of MC were significantly higher among samples obtained from MB than those from NG (Mann–Whitney test, *p* < 0.001; Figure 4A). In MB, the MC concentrations showed a decreasing gradient from the inshore (0.15–11.7; mean ± SD = 2.01 ± 2.37 µg MC-LR equiv. L^−1^) and recreational stations (0.18–14.8; 2.34 ± 4.03 µg MC-LR equiv. L^−1^) to the WTP abstraction point (0.04–1.41; 0.62 ± 0.47 MC-LR equiv. L^−1^; ANOVA, *p* = 0.0128) and open lake stations (0.02–0.91; 0.22 ± 0.25 MC-LR equiv. L^−1^, ANOVA, *p* < 0.05). In NG, the mean MC concentrations were rather low (<0.2 µg MC-LR equiv. L^−1^), with no significant difference across the sampling stations (ANOVA, *p* = 0.904; Figure 4A). In MB, MCs were detected throughout the year, but in NG, MCs mostly occurred during the first half of the study period, i.e., November 2017–April 2018 (Figure S3).

**Figure 5:**
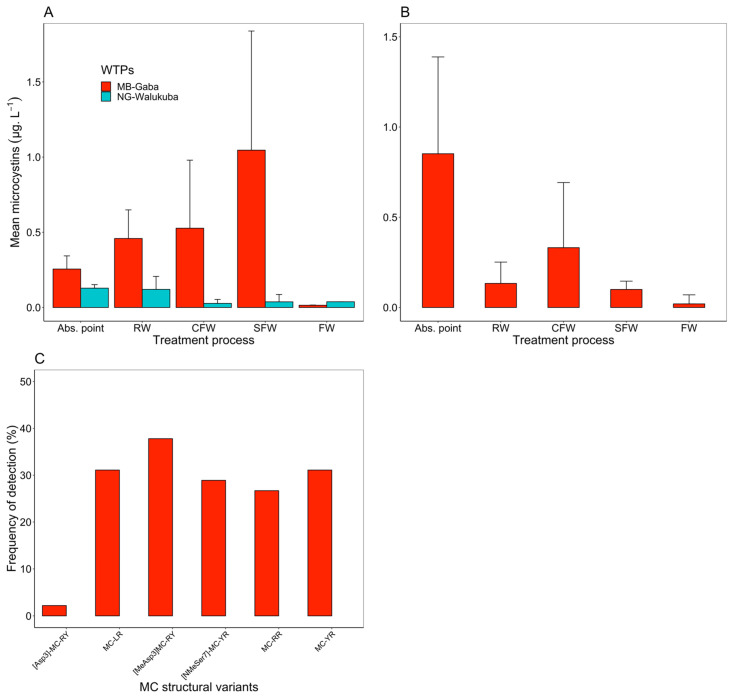
Concentration of dissolved MC (mean ± SD) as detected by ELISA (**A**) and LC-MS (**B**) from WTP abstraction points in MB and NG, and during the treatment process in MB-Gaba and NG-Walukuba. (**C**) Relative frequency of the structural variants of MC detected in the dissolved water from MB-Gaba (*n* = 45). Note: there was no detection of dissolved MC in NG-Walukuba. Abbreviations: Abs. point = WTP abstraction point, RW = Raw water, CFW = Coagulated and flocculated water, SFW = Sand filtered water and FW = Final water.

*During water treatment:* During the water treatment survey, there was no analysis for ATX (HTX), CYN and STX. In total, 21 samples (23.3%, *n* = 90) were found positive for MC. For the MB-Gaba abstraction point and raw water, six MC congeners, including MC-RR, MC-YR, MC-LR, [Asp^3^]-MC-RY, [MeAsp^3^]-MC-RY and [NMeSer^7^]-MC-YR, were regularly detected. In NG-Walukuba, four congeners were detected at the abstraction point, including MC-LR, MC-RR, MC-YR and [NMeSer^7^]-MC-YR (Figure 4D). For the WTP survey, both intracellular and dissolved MCs were differentiated. In general, intracellular MC concentrations at the two WTP abstraction points were found comparable to MC concentrations observed inshore (Figure 4B), i.e., the mean concentrations of intracellular MCs were 1.22 ± 0.36 µg MC-LR equiv. L^−1^ at MB-Gaba and 0.36 ± 0.46 µg MC-LR equiv. L^−1^ at NG-Walukuba. For MB-Gaba only, the MC concentration increased to 3.61 ± 0.48 µg MC-LR equiv. L^−1^ in raw water (Figure 4B). Accordingly, the concentrations of dissolved MCs, as determined via ELISA, were significantly higher in MB-Gaba than in NG-Walukuba (Figure 5A). However, for NG-Walukuba, the dissolved MCs were consistently below the LOD for LC-MS (Figure 5B). In addition, there was an increase in dissolved MC during certain treatment steps in MB-Gaba, especially coagulation and flocculation, but in general, the dissolved MC was drastically decreased until the final treatment step. Indeed, MC was detected in the final treated water only once (0.14 µg L^−1^ on 4 January 2017). In the diluted fraction, MCs were composed of MC-LR, MC-YR, [MeAsp^3^]-MC-RY and [NMeSer^7^]-MC-YR (Figure 5C, Tables S1 and S2).

### 2.3. Relationships between Predictive Variables and Microcystins

Regarding the limnological variables selected to predict MC concentration (Table 1), turbidity ranged from 0.2 to 197 NTU, and the dissolved N:P ratio ranged from 0.16 to 149.1 (mean 18.2 ± 22.3). Among the biological variables, the average biovolumes and cell numbers of *Microcystis* were higher than *Dolichospermum*. The meteorological variables, such as rainfall, during the sampling days varied from no rain (dry season) to maximum rainfall of 61.1 mm/day and an average of 11 mm for the sampling period. The primary wind directions were from north to south (N–S), from east to west (E–W) and from northeast to southeast (NE–SE). In particular, during the field survey period (November 2017–October 2018), 30.4% of the sampling days had an average wind speed of 4.3 km h^−1^ in the N–S direction, followed by 26.1% of sampling days with a wind speed of 3.5 km h^−1^ in the E–W direction and 21.7% days with wind of 5.4 km h^−1^ in the S–SE direction. The average wind speed ranged from 3.1 to 8.1 with a mean of 4.5 ± 1.1 km h^−1^, while the mean solar radiation (for the 10 days prior to each sampling date, D10) was 291 ± 81 Wm^2^ day^−1^.

Taking all the data together (*n* = 120), the parameters of wind speed and wind direction, mean intensity of solar radiation and biovolume of *Microcystis* and *Dolichospermum* explained 55.2% of the variance of the partial least squares (PLS) model for MC concentrations (PLS model coefficients not shown). When considering the cell numbers of biological parameters in the PLS model, wind speed, direction, mean solar radiation and *Microcystis* cell numbers explained 49.6% of the variance in MC concentrations (Table 1). Furthermore, there was a significant correlation between *Microcystis* in MB and MC concentration (abundance data: r = 0.57, *p* < 0.001; biovolume data: r = 0.62, *p* < 0.001), but no significant correlation between *Dolichospermum* (abundance or biovolume) and MC concentration.

**Figure 6:**
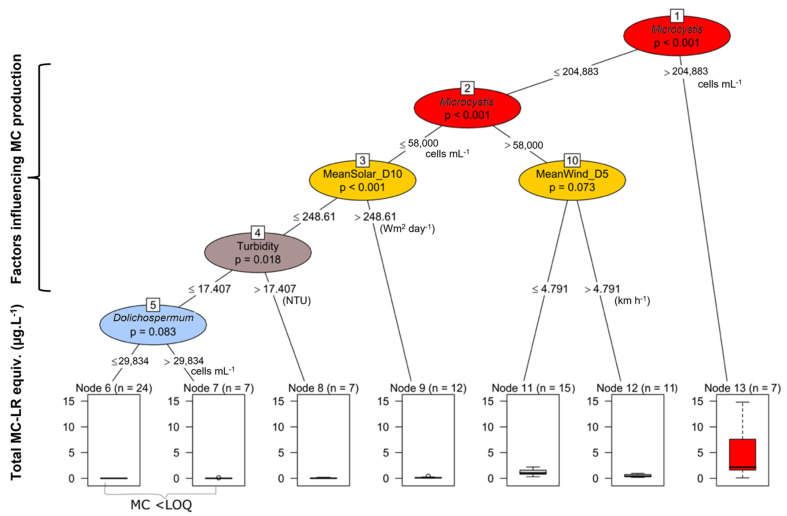
Decision support tree for prediction of MC concentrations in lake water from Murchison Bay and Napoleon Gulf, northern Lake Victoria. Note: LOQ for MC = 0.04 µg L^−1^; MeanWind_D5 = the mean wind speed for the five days (km h^−1^) (N–S direction) and MeanSolar_10 = mean solar irradiance for the ten days prior to sampling (W m^2^ day^−1^). (Data collected between November 2017 and October 2018, *n* = 120). Colors: Red = 0.08–14.8 µg MC-LR equiv. L^−1^, Yellow = LOQ–4.5 µg MC-LR equiv. L^−1^, Brown and Blue = LOQ–0.91 µg MC-LR equiv. L^−1^.

The decision support tree (DST), which is a guiding tool for management actions during suspected harmful blooms, was applied to provide an alert level framework for the health risk through MC concentrations as well as the need to monitor MC concentrations. As a precautionary tool, in the DST, the prediction of MC was primarily related to the abundance of *Microcystis* spp. (support vector machines with linear kernel, kappa = 0.7877, *p* < 0.001; Figure 6). When *Microcystis* abundance was >200,000 cells mL^−1^ (or equivalent of 13.7 mm^3^ L^−1^), there was a 100% probability of observing MC. When *Microcystis* abundance was between 58,000 (the equivalent of 3.9 mm^3^ L^−1^) and 200,000 cells mL^−1^, the MC concentrations were significantly related to the mean solar radiation (>248 Wm^2^ day^−1^) and mean wind speed (<4.8 km h^−1^) in the N–S direction. Additionally, when *Microcystis* abundance was <58,000 cells mL^−1^, the mean solar radiation intensity for the 10 days prior to sampling (D10), turbidity (>17.4 NTU) and abundance of *Dolichospermum* were correlated with low concentrations of MC (Figure 6).

## 3. Discussion

### 3.1. Toxigenic Cyanobacteria and Cyanotoxins in the Lake Victoria Embayments

The predominant toxigenic cyanobacteria in the two embayments of northern Lake Victoria during the study period were *Microcystis* in MB and *Dolichospermum* and *Microcystis* in NG. These two genera are among the most common toxigenic genera in aquatic freshwater ecosystems, followed by *Raphidiopsis* (previously *Cylindrospermopsis*) [5]. The *Microcystis* biovolume recorded between April 2003 and March 2004 formed 0.3 ± 0.1 mm^3^ L^−1^, representing 20.1% of the total cyanobacteria biovolume (1.6 ± 0.7 mm^3^ L^−1^) in inner MB [20]. In another study between May and June 2004, the *Microcystis* biovolume ranged from 3.1 to 23.2 mm^3^ L^−1^ [21], and in 2017–2018 (this study), it varied between 0.5 and 69 mm^3^ L^−1^ (10.5 ± 10.6 mm^3^ L^−1^). Considering these data, they suggest that there has been an increase in *Microcystis* biomass (six-fold) in MB over the last two decades.

Analogously, the range of *Dolichospermum* biovolume in the mid-2000s ranged from 8.6 to 15 mm^3^ L^−1^ [21], but during this study, it varied between 0.4 and 20.6 mm^3^ L^−1^. In NG, the *Microcystis* biovolume varied between 3.1 and 23.2 mm^3^ L^−1^ in the mid-2000s and 0.18 and 31.4 mm^3^ L^−1^ (this study), but the *Dolichospermum* biovolume declined from 9.8–83 mm^3^ L^−1^ to 0.1–8.63 mm^3^ L^−1^. This suggests that either during the mid-2000s, a *Dolichospermum* bloom event occurred in NG, or the dominance of *Dolichospermum* shifted to *Planktolyngbya* more recently. This shift between the two genera, i.e., heterocyte-forming *Dolichospermum* vs. non-heterocyte-forming *Planktolyngbya*, could reflect the increased inorganic nitrogen inputs, i.e., via terrestrial run-off.

Our study also revealed the presence of HTX at the inshore station of MB. This neurotoxic cyanotoxin is known to be produced by cyanobacteria from the genera *Dolichospermum*,* Aphanizomenon*,* Oscillatoria*,* Phormidium* and *Planktothrix* [22]. In this study, among the potential HTX producers, at the same time, *Dolichospermum* was observed in the depth-integrated water sample; thus, the detected HTX was possibly associated with this genus. For instance, the *Dolichospermum* biovolumes were 8.6 and 5.1 mm^3^ L^−1^ during June and September 2018 when HTX were detected from the inshore areas in MB. On the other hand, in this study, *Oscillatoria tenuis* was detected once in November 2017 but with high abundance. *Oscillatoria* can either produce ATX only or HTX as a major variant with traces of ATX (see Mann et al. [23]). In the future, the suggested HTX occurrence in Lake Victoria embayment should be evaluated by cyanobacteria strain isolation.

The main cyanotoxins detected in lake water and during water treatment included MCs with the congeners MC-RR, MC-YR, MC-LR, [Asp^3^]-MC-RY, [MeAsp^3^]-MC-RY and [NMeSer^7^]-MC-YR, which have been recorded already in previous studies in these embayments, as well as partly from other parts of the lake (see review [8]). However, while the congeners were similar, in this study, for the MB embayment, the MC concentrations were higher than those reported previously (0.2–15 µg MC-LR equiv. L^−1^ in this study compared to 0.2–0.7 µg MC-LR equiv. L^−1^ recorded during the mid-2000s (particularly from 2004 to 2005) [24]; and 0–1.6 µg MC-LR equiv. L^−1^ between 2007 and 2008 [13]). This increase in MC concentrations might be associated with the six-fold increase in the mean *Microcystis* biovolume (10.5 ± 10.6 mm^3^ L^−1^) in MB. On the contrary, in NG, the concentrations of MC reported in this study corresponded to those reported in the late 2000s, estimated between 0 and 1.5 µg MC-LR equiv. L^−1^ [13,21,25]. Obviously, the change in cyanobacterial community composition from *Dolichospermum* dominance to *Planktolyngbya* dominance did not affect MC concentrations in NG, since this genus does not produce MC [26].

The concentrations of the MC detected in Lake Victoria have varied over the past two decades. In general, the concentrations of MC in the water column (depth-integrated samples) have been found relatively low (≤2 µg L^−1^, e.g., in Ref [8]). However, during bloom events and in surface sums, high concentrations of 81 µg L^−1^ [27] and >2 mg L^−1^ [12] have been reported in Nyanza Gulf (Kenya). This implies that higher concentrations of MC in the lake can occur, as well as the consequences for humans and fish. In Africa, MCs have been detected in 21 of the 55 countries, where in the south, the concentrations have ranged between <1 and <20 μg L^−1^ in Zimbabwe (and up to 43 µg L^−1^ in Lake Krugersdrift in South Africa [28]). Similarly, in the west and north African countries, MC concentrations ranged from 3.2 µg L^−1^ (Ghana), 5.8 µg L^−1^ (Nigeria) and up to 28.9 µg L^−1^ (Algeria) [28]. This can affect direct water use and even wildlife, as reported from the Kruger National Park, when MC concentrations exceeded > 2 mg L^−1^ [29]. Consequently, the reported MC concentrations can have variable consequences for humans, livestock and wildlife on the African continent.

### 3.2. Factors Explaining MC Concentrations

The most important drivers explaining MC concentrations are cyanobacterial species composition, followed by physiological factors related to cellular growth, such as light intensity and rising temperature [30], and the concentrations and forms of N and P [31,32]. For various lakes in Uganda, Poste et al. [24] showed that high TP, low TN:TP ratios and high cyanobacteria biomass all positively influenced MC concentrations. Recently, Krausfeldt et al. [33] indicated that although N sustained the biomass of toxigenic cyanobacteria, different forms of N can induce physiological changes, consequently causing variations in the MC concentrations. Aside from the physiological effects, all these drivers are triggers of growth of the toxigenic cyanobacteria, *Microcystis* and *Dolichospermum*, and thus also influence the production of MC through cell division [34]. Earlier studies by Roegner et al. [35] did not find potential predictors of MC presence or an increased MC concentration in another embayment, Kisumu Bay, Lake Victoria (Kenya). During our study period, the variation in MC concentration was associated with the biovolume of *Microcystis* spp. and mean solar radiation, while changes in mean wind speed and direction (N–S and N–NE) were also related to the incidence of toxigenic *Microcystis* sp. and MC in the two embayments. From the decision tree, which provided a stepwise increase in MC concentration (Figure 6), it can be inferred that MC concentration was increasing at lower wind speed (<4.8 km h^−1^) blowing in the N–S direction, suggesting that the wind was blowing the scums from the inshore to the open water. In general, wind-driven currents are important in the horizontal and vertical distribution (sinking or floating) of *Microcystis,* which depend on the colony size [36] and physical density of the *Microcystis* cells [37]. During the study period, the most frequent wind direction was N–S oriented (30.4%), i.e., moving surface water from the inshore areas to the open lake. In contrast, the open lake had less chance of being blown into the bays (S–N) and to the shorelines (S–NE), since only 4.3% of the sampling days had wind blowing in these directions. Thus, the southward wind might have influenced the movement of *Microcystis* scum from the inshore areas to the open water and thus reduced MC concentrations.

In general, it is known that *Microcystis* growth is favored by eutrophic and polymictic conditions because *Microcystis* form buoyant colonies that can float up quickly and use the light for photosynthesis more efficiently than other non-buoyant taxa, such as diatoms [38]. For example, in Nyanza Gulf of Lake Victoria (Kenya), the growth of the chain-forming diatom *Aulacoseira granulata* was found positively related to transparency (Secchi depth), while the growth of *Microcystis* sp. was found negatively related [39]. Marshall et al. [40] predicted an overall increase in temperature by 2 °C in northern Lake Victoria by 2055, which will also cause increased physical stratification of the main basin and possibly favor the growth of buoyant cyanobacteria, such as *Microcystis.*

In addition, an earlier field survey from MB and NG and other Ugandan water bodies [21] suggested that the concentration of MC per *Microcystis* cell was causally dependent on the quantity of MC-producing genotypes, i.e., those carrying the *mcy*B gene, which is indicative of MC synthesis. The same authors also reported a relationship between MC concentrations and total *Microcystis* cell numbers, which was also found in this study. It was reported that for individual strains, high light availability increased MC synthesis in *M. aeruginosa* by increasing gene transcription [41]. Under high light intensity, the higher level of MC was related to the higher transcription levels of both *mcy*B and *mcy*D genes [42]. In this study, neither the genetic basis nor genetic regulation of MC biosynthesis was tested, but in addition to *Microcystis* biovolume, accordingly, the effect of light availability, i.e., through lower turbidity (or increased transparency), and average solar radiation (a proxy for both light and temperature) were found positively related to MC concentration.

### 3.3. The Fate of MC during Water Treatment

In general, WTPs are susceptible to cyanotoxin contamination, but the data on cyanotoxin occurrence in treated water produced in the tropics are scanty [4,43]. Earlier studies in Lake Victoria focused on intracellular toxins [8,34], but fewer considered extracellular toxins. This study monitored both intra- and extracellular MC occurring through a conventional water treatment installed at the two embayments susceptible to cyanobacterial blooms. Chorus and Bartram [43] reported that WTPs in general, using coagulation, clarification and rapid sand filtration for surface water treatment, are effective at removing cyanobacterial cells but only partially effective at removing free (dissolved) cyanotoxins. Indeed, rapid sand filtration can be considered effective in removing intracellular MC through the removal of cyanobacterial cells. For instance, in this study, chlorophyll-a concentration recorded in raw water (47.6 ± 7.7 µg L^−1^ in Gaba III and 12.3 ± 5.4 µg L^−1^ in Walukuba) declined by >90% in final water (i.e., 2.7 ± 1.8 µg L^−1^ in Gaba and 2.3 ± 0.4 µg L^−1^ in Walukuba), suggesting that sestonic algae and cyanobacterial cells were effectively removed. In addition, our data showed that intracellular MCs were detected in 62.6% and 33.3% of the raw water samples from MB-Gaba III and NG-Walukuba, respectively, but were no longer detected in final water.

On the other hand, rapid sand filtration may be more susceptible to cell lysis [4,43]. Therefore, the water authorities in charge of conventional WTPs might face a challenge arising from dissolved MC. In this study, when raw water MC concentration was 0.46 ± 0.19 µg MC-LR equiv. L^−1^, we observed dissolved MC during rapid sand filtration. This is in agreement with studies in Finland, where Tarczynska et al. [44] found that, on average, 0.8 µg L^−1^ of MC was detected in treated water, suggesting that pre-chlorination, coagulation and rapid sand filtration were ineffective in removing dissolved MC. Similarly, Lahti et al. [45] also reported that rapid sand filtration was less effective in removing dissolved MC due to the occasional occurrence of MC in treated water below the lifetime guideline value for exposure via drinking water (WHO 2021). As a result, the release of MC from cells should receive more attention during water treatment via rapid sand filtration. For effective removal of dissolved MC, an additional process is required, e.g., Ho et al. [46] reported complete degradation of MC on biologically active sand filters within four days, attributed to high bacterial activity. Another effective method to remove dissolved MC was slow sand filtration or filtration through granular activated carbon [47]. However, both methods are less common in water treatment in east Africa and the global south.

For MB-Gaba, the dissolved MC detected in the sand-filtered final water was possibly associated with filter clogging and more frequent backwashing per day (min. 2 times per day). It is possible that a higher cyanobacteria biomass in the abstracted raw water caused a higher dissolved MC concentration. Nevertheless, the total dissolved MC concentration in treated water was always found below the lifetime guideline value for exposure via drinking water (WHO 2021, i.e., <1.0 µg MC-LR equiv. L^−1^). However, since an increase in *Microcystis* biovolume in raw water in the future cannot be excluded, regular monitoring of dissolved MC during the water treatment process is recommended to better understand the potential health threats over longer periods. This will be in addition to the daily measurements of pH, conductivity, water color, turbidity, total suspended solids, and residual chlorine, and *E. coli* measurements for treated water safety for the riparian population.

### 3.4. Multiple Exposure Routes to Toxigenic Cyanobacteria and Cyanotoxins

The primary threat to humans is via direct exposure to toxigenic cyanobacteria and cyanotoxins through drinking water [4], mainly from contaminated untreated or treated water [34]. In our study, the two WTPs efficiently purified raw water containing toxic cyanobacteria. However, probably through cell lysis (after pre-chlorination and coagulation), free intracellular MCs were released into the water and were composed of five or six structural congeners. In addition to the globally most frequent MC-RR, -YR and -LR variants, the rare [NMeSer^7^]-MC-YR congener was found frequently. It should be mentioned that chlorination of raw water or treated water has been reported to be associated with the formation of undesirable degradation by-products and isomers, such as monochloro-MC, monochloro-dihydroxy-MC and tri-halo-menthanes [48]. Thus, in the future, if an increased concentration of free MC is recorded in final water, investigations on the potential chlorination degradation by-products would be required as well.

In general, MC-LR was found to be the most potent toxin, followed by MC-YR and MC-RR, i.e., when administered intraperitoneally to mice (e.g., Ref [49]). When administered orally to mice, fewer indications of acute toxicity have been found for two MC-RR related structural variants (e.g., Chernoff et al. [50]). To our knowledge, the toxicity equivalents for the more frequently detected [NMeSer^7^]-MC-YR and MC-RY variants during the WTP survey were not determined. However, since MC-LR was recorded frequently during both the field survey and the WTP survey, the resulting toxicity is considered relevant. It should be noted that, in the absence of oral toxicity data for other MC structural variants, it is recommended that the guideline values are applied to total MCs, based on the worst-case assumption of the congeners having similar toxicity [4]. In general, the average intracellular MC concentrations observed inshore exceeded the lifetime (1.0 µg MC-LR equiv. L^−1^) guideline value in MB but not in NG. Therefore, to guarantee the elimination of MC from treated water, the actual toxin concentrations in the source water should be monitored. Early warning parameters, such as *Microcystis* cell numbers or pigments, could be used to trigger an action plan for safety during water treatment if thresholds of toxigenic cyanobacteria and cyanotoxins in the source water are exceeded.

In addition, for Uganda, the use of untreated water by local populations engaging in recreational activities is considered an important exposure route. For example, in this study, the highest concentration of MC in MB (15 µg MC-LR equiv. L^−1^ recorded at the recreational areas) coincided with a *Microcystis* cell density of 1.45 × 10^6^ cells mL^−1^. Thus, the nearshore recreational areas, which are easily accessible by the locals, expose them to toxic cyanobacteria and cyanotoxins. In general, the observed *Microcystis* cell densities (*M. flos-aquae*, 859,690 ± 582,749 cells mL^−1^ and *M. aeruginosa*, 792,609 ± 418,230 cells mL^−1^) exceeded the frequently cited alert level of 100,000 cells mL^−1^ when assessing health risk and regular monitoring is recommended [43]. A general increase in MC-producing *Microcystis* in MB would suggest that the probability of exceeding the WHO lifetime guideline value of 24 µg MC-LR equiv. L^−1^ in recreational water (untreated water) will increase as well. Thus, the monitoring of *Microcystis* cell abundance could be used as an early warning tool both for recreational services providers and WTP operators [51]. Consequently, measures to reduce the threats of deliberate and accidental consumption of toxigenic cyanobacteria during recreation, and eventually, adjustments in the dosages of the chemicals used in the water treatment process, are recommended [52]. These measures could involve warning signs and the closure or discontinuation of swimming or bathing. So far, these measures have been effective in developed countries, such as the Netherlands [52], but have not been used in the south, including Uganda. For WTP operation and due to seasonal variability in toxigenic cyanobacteria and the high cost of toxin monitoring, it is deemed necessary to adopt simple, rapid methods, such as cell counting of the dominant toxigenic taxa of *Microcystis*.

## 4. Conclusions

This study is the first to examine the seasonal and spatial diversity and the dynamics of MC in lake water used for water treatment in Murchison Bay and Napoleon Gulf in Lake Victoria. Although MCs were efficiently reduced by both WTPs, the high abundance of toxigenic cyanobacteria within the lake water and the possible occurrence of the cyanotoxin MC can pose a potential health threat to riparian communities. The risk of daily consumption of untreated water by locals and people engaging in recreation requires an early warning system, especially during cyanobacterial bloom events. The increase in MC concentration was related to the biovolume of *Microcystis*, influenced through solar radiation, mean wind speed (N–S direction) but also turbidity in the water column. Thus, early warning methods, such as regular *Microcystis* biovolume estimation and sensitization of people during bloom events, are proposed to prevent health threats from toxigenic cyanobacteria. According to the results of this study, we recommend not using lake water for domestic purpose (cooking, drinking, washing) but rather using treated water and reducing recreational activities during *Microcystis* bloom events.

## 5. Materials and Methods

### 5.1. Study Sites and Sampling Design

The two bays of Lake Victoria, Murchison Bay (MB) and Napoleon Gulf (NG), were selected for this study because of their contrasting ecological conditions and contrasting abundances and diversity of toxigenic cyanobacteria [19] (Figure 1). MB is a relatively shallow (maximum sampled depth = 18 m) and closed bay, receiving mainly point source pollutants, while NG, although similarly shallow (18.1 m), is an open gulf and hosts the major outflow from Lake Victoria (source of River Nile). These bays are the main water source for local inhabitants who are not connected to piped (treated) water using lake water processed by the WTPs.

Depending on the location, the sampling depth ranged from 1.5 to 18 m (Figure 1). At the various sampling stations, water samples were depth integrated from every 1 m depth using a 2 L horizontal van Dorn sampler. The samples were collected in a 20 L bucket as a composite sample to represent the whole water column. From the depth-integrated sample, 2 L were taken and transported in cooling boxes to the laboratory for filtration using GF/C filters under low vacuum pressure for cyanotoxin analysis (see below).

In order to estimate cyanobacteria biovolume composition and MC during water treatment, sampling was performed weekly (*n* = 9) between November 2016 and January 2017 at the Gaba III plant (MB-Gaba) located in MB and at the Walukuba plant (NG-Walukuba) in NG (Figure 1). Although both plants use the conventional water treatment methods of coagulation, rapid sand filtration and chlorination, differences exist between the capacity of the WTPs and additional treatment steps applied in the Gaba III plant. The main differences are that MB-Gaba III abstracts raw water about 4100 m^3^ h^−1^ from 1.5 km offshore at a depth of 8 m, while NG-Walukuba abstracts about 880 m^3^ h^−1^ from 600 m offshore at a depth of 4 m. In addition, the MB-Gaba plant performs pre-chlorination of the raw water and pH adjustment of the final treated water by using calcium carbonate (CaCO_3_). Sampling was performed to monitor the intracellular and dissolved MC concentration during different steps of the water treatment: (i) in the lake water at the WTP abstraction point, (ii) in the raw water at the entry point of the WTP, (iii) after flocculation and sedimentation, (iv) after sand filtration, (v) in final water after chlorination (Table 2). In general, the abstraction pipes are protected with a mesh to prevent the uptake of course matter. After abstraction, the water is pumped to the WTP for treatment, usually within a short retention time (the pumped water is considered as raw water used for water treatment).

### 5.2. Meteorological, Physical-Chemical and Biological Variables

Daily meteorological data (rainfall, air temperature, wind speed and solar radiation) for October 2017 to October 2018 were obtained from the Jinja and Kampala weather stations operated by the Uganda National Meteorological Authority (UNMA) and are described in Olokotum et al. [19]. In addition, wind direction data for the same period were acquired from https://www.timeanddate.com (retrieved on 12 January 2022).

For each sampling date in the lake, several physical-chemical variables (water depth, Secchi depth, temperature, pH, electrical conductivity, dissolved oxygen and turbidity measured in situ) and soluble reactive phosphorus (SRP), nitrate (NO_3_-N), nitrite (NO_2_-N), ammonium (NH_4_-N), soluble reactive silica (SRSi), total phosphorus and total nitrogen were measured at the analytical laboratory of the National Fisheries Resources Research Institute (NaFIRRI), following standard operating procedures, as described in Olokotum et al. [19].

For microscopical analysis, 20 mL of the water samples were fixed using Lugol’s solution [53], stored away from light. Later, 2–5 mL of the fixed water samples were sedimented for 6–12 h [53] before taxa identification and enumeration. Cyanobacteria taxonomic identification and taxa abundance and biovolume estimation were performed based on geometric shapes described in Sun and Liu [54]. The cyanobacteria species were identified using a taxonomic guide and keys as described in Talling [55], Komárek and Anagnostidis [56,57,58], and Cronberg and Annadotter [59]. Chl-a concentration was used as a guide to estimate the volume of sample that should be sedimented. Total phytoplankton biovolume was positively correlated with Chl-a (R^2^ = 0.55, *p* < 0.001) [19]. When Chl-a concentration was low (≤20 µg L^−1^), 5 mL was sedimented. When it was high (≥20 µg L^−1^), 2–3 mL was sedimented. In addition, *Microcystis* was first counted as colonies from which the number of cells per colony was estimated based on the size of the colony and cell size. Later, the samples containing *Microcystis* sp. were re-counted using a Malassez counting chamber to estimate the *Microcystis* cell numbers.

### 5.3. Cyanotoxins Analysis

#### Field Survey (Quantification of Intracellular MC)

For intracellular cyanotoxins analysis, the filtered biomass was extracted using 75% aqueous methanol, according to the protocols described by Cerasino et al. [60], with slight modifications. Briefly, the cultures and environmental samples were thawed to room temperature in sterile 15 mL glass vials and later conditioned with 4 mL of 75% methanol. The conditioned samples were sonicated on ice using an ultrasound probe (Sonics Vibra cell) (100% amplitude, 130 W, 20 kHz) for 3 min with 30 s interval pulses to aid extraction. The extracts were centrifuged at 4,000 rpm for 15 min (4 °C), and the supernatant was collected in clean 15 mL glass vials. An amount of 2 mL of 75% methanol was added to the pellet for the second extraction using an iced ultrasonic bath (Prolabo) (100% ultrasound power at 40 kHz) for 15 min. The second extracts were also centrifuged and supernatants pooled together. Later, 2 mL of the pooled extract was centrifuged at 13,000 rpm for 10 min, and part of the supernatant extracts was stored in 2 mL amber glass vials at −20 °C until MS analysis. For ATX, CYN, STXs and MC analysis, cyanobacteria strain cultures of *Phormidium favosum* (PMC 240.05), *Raphidiopsis raciborskii* (PMC 99.03), *Aphanizomenon gracile* (PMC 638.10) and *Microcystis aeruginosa* (PMC 728.11), respectively, were used as positive controls (Table S4).

For the characterization and quantification of intracellular MC by LC-MS/MS, the extracts were centrifuged at 13,000 rpm for 10 min, and 500 µL of the clear supernatant was transferred into HPLC glass vials. Four microliters werre injected into an ultra-high-performance liquid chromatography (UHPLC) chromatographic chain (Elute UHPLC-Bruker) coupled to a high-resolution mass spectrophotometer (MS) system (Compact QTOF-Bruker) for identification and quantification of the target cyanotoxins. The extracts were separated on a grafted-C18 stationary phase column (Acclaim RSLC polar advance 2, 2.2 μm, Thermo Fisher^®^, Waltham, MA, USA, 2.1 × 100 mm) along a 15 min linear gradient (5–90%; 0.3 µL min^−1^ flow rate) of ACN containing 0.08% formic acid and ultrapure water acidified with 0.1% formic acid. In the MS, the cyanotoxin masses were analyzed between 50 and 1500 *m*/*z* in broad-band collision ion dissociation (bbCID) or auto MS-MS/MS positive mode, alternating at 2 Hz the MS and MS2 modes at low and high energy, respectively (mass accuracy of <0.5 ppm).

The cyanotoxins were identified according to (i) retention time, (ii) molecular mass, (iii) isotopic pattern and (iv) diagnostic ions. They were quantified according to the area-under-the-peak signal determined for analytical standards of STX (Cas 35554-086), ATX (Cas 64285-06-9), HTX (Cas 14926-86-1), NOD (Novakit^®^), CYN (Novakit^®^) and seven variants of MC (MC-LR, -LA, -LF, -LW, -LY, -RR and YR) (Novakit^®^) using the TASQ software v1.3 (Bruker^®^, Bremen, Germany). Except for MC-RR, MC-LR and MC-YR, the other MC structural variants were quantified as MC-LR equivalents calculated from the regression curves of the MC-LR analytical standard. The LOD and LOQ for the MC standard were 0.02 µg L^−1^. The HTX was also identified according to (i) retention time, (ii) molecular mass, (iii) isotopic pattern and (iv) diagnostic ions previously determined by analyzing a commercial standard (Abraxis) in the same platform and under the same conditions (RT = 4.0 min, transition ion list *m*/*z* 180 --> 163; *m*/*z* 180 --> 145; *m*/*z* 180 --> 135; *m*/*z* 180 -->107).

### 5.4. Abstraction Points and Water Treatment (Quantification of Intracellular MC)

Intracellular MCs were extracted according to Fastner et al. [61]. In brief, the filters carrying the algal biomass were cut into small pieces, and the algal biomass was extracted in aqueous 75% (*w*/*v*) methanol (1.5 mL volume). The extracts were sonicated in a water bath (Bandelin Sonorex Ultrasonics) for 10 min and then transferred to a shaker for 30 min. Subsequent to centrifugation at 13,000 rpm (10 min), the clear supernatant was transferred into new 2 mL reaction tubes, which were evaporated to dryness in a vacuum concentrator at 30 °C. The procedure was repeated two times to ensure efficient MC extraction.

For liquid chromatography–mass spectrometry (LC-MS) analysis, the dried extracts were resuspended using 150 µL of 100% (*v*/*v*) methanol, sonicated for 10 min, and 150 µL of MilliQ (MQ) water was added. The clear extracts were then injected into the HPLC-DAD using a LiChrospher ^®^ 100, octyldecyl silane (ODS), 5 µm particle size, LiChroCART ^®^ 250-4 HPLC cartridge system (Merck, Darmstadt, Germany). According to Lawton et al. [62], for chromatographic separation, a linear gradient of aqueous acetonitrile (ACN) in 0.05% Trifluoroacetic acid (TFA) (30–70% ACN) for 45 min was used. The different variants of MCs were identified by (i) retention time and the order of elution using the analytical MC standards (MC-RR, YR and LR) and (ii) protonated mass [M + H]^+^ and MS^2^ and MS^3^ fragmentation patterns using an iontrap (amaZon SL, Bruker Daltonics, Bremen, Germany). The LOQ for the MC standards were 0.05 µg injected. The globally rarer structural variants of MC [Asp^3^]-MC-RY, [MeAsp^3^]-MC-RY and [NMeSer^7^]-MC-YR were described from the same habitats previously [21]. Representative LC-MS chromatograms are presented as Supplementary Material (Figure S5).

The UV and MS spectra and peaks were manually integrated to obtain the peak area from which the MC concentrations were determined, which were quantified as concentration equivalents of external analytical standards MC-RR, YR and LR (Cyano Biotech GmbH, Berlin, Germany). These calibration curves of the MC structural variants were pooled together, and the total MC concentration was calculated from the regression curve—y = 1626.7x + 0.0989 (R^2^ = 0.99), where y was the absorption (mAU) recorded at 240 nm wavelength (UV), and x was the concentration of the MC standards injected in the column.

### 5.5. Abstraction Points and Water Treatment (Quantification of Dissolved MC Using ELISA)

All water samples were transported cool and dark within 2–4 h to the laboratory for filtration. Depending on the turbidity from the sample, 250–1000 mL was filtered through the Whatman GF/C filters (Ø 47 mm) using a low vacuum pump. For the MB-Gaba and NG-Walukuba samples, both intracellular (filtered biomass) and dissolved MCs (filtrate) were determined. For the analysis of dissolved MCs in the filtrate, we directly used the Abraxis MC ELISA Kit (Product No. 520011, Biosense Laboratories, Bergen, Norway). The absorbance at 450 nm was determined using an ELISA plate reader (LEDETECT 96, SN: 1357) in duplicate within 15 min. Standard calibration curves were constructed using the MC-LR standard (150, 400, 750, 1000, 2000 and 5000 ng L^−1^) from which the concentrations of total dissolved MC in the samples were calculated in the linear range (0.2–2 ng/mL of MC-LR equiv.) according to the manufacturer’s instructions.

### 5.6. Abstraction Points and Water Treatment (Quantification of Dissolved MC Using LC-MS)

The same GF/C filtrated samples were concentrated through C18 columns (Sep-Pak ^®^ Vac tC18 cartridge, 1 cc/100 mg, 37–55 µm, Waters Corporation, Vienna, Austria) using a standard solid phase extraction (SPE) procedure according to Dean [63]. We tested the SPE protocol at the Research Institute for Limnology, Mondsee, using 100 mL of lake water (Lake Mondsee), tap water and Millipore water spiked with 4 µL of analytical MC standards (MC-RR, MC-YR and MC-LR with MC-LR concentration equivalent to 1.0 µg L^−1^). Briefly, C18 columns were conditioned using 4.0 mL of 80% (*v*/*v*) methanol and equilibrated using 1.0 mL of MilliQ (MQ) water. Thereafter, 500–700 mL of the filtrate was allowed to flow through the column at 3 drops/second. For a pilot test, half of the columns were eluted immediately (wet), while the other half were dried at 50 °C for 48 h. Since no significant difference in the recovery of dissolved MC was observed, subsequently, SPE columns were dried at 50 °C for 48 h and, thereafter, stored frozen at −20 °C. SPE columns were transported cool and dry to the laboratory in Mondsee, Austria. In the laboratory, the C18 SPE columns were eluted with 1.0 mL of 80% (*v*/*v*) methanol. The eluted volume (1 mL) was concentrated and injected into the HPLC-DAD (HP1100, Agilent, Vienna, Austria) coupled to mass spectrometry (MS, amaZon SL, Bruker, Bremen, Germany). This protocol resulted in an overall acceptable recovery of MC-RR, MC-YR and MC-LR analytical standards (CyanoBiotech GmbH, Berlin, Germany) (Figure S4). During the WTP survey (November 2016 to January 2017), SPE samples (filtrates) from the lake and the subsequent steps in water treatment were also spiked using 4 µL of the analytical MC standards with MC-LR concentration equivalent to 1.0 µg L^−1^. All the analytical MC structural variants (MC-RR, YR and LR) were recovered from C18 SPE columns with concentrations > 1.0 µg L^−1^ (Table S3).

### 5.7. Statistical Data Analysis

For cyanobacteria, the taxa were grouped into two classes of (i) (potentially) toxigenic cyanobacteria (taxa that were already described as toxin producers using strains) and (ii) non-toxigenic cyanobacteria (if toxin synthesis was not described from strains) [25]. The data on cyanobacteria biovolume composition were pooled for MB and NG and then compared using the Mann–Whitney test. Within NG, the differences between the sampled stations were compared using repeated-measures ANOVA, while within MB, a non-parametric test (Friedman test) was performed.

During the water treatment processes, the efficiency of the treatment step was estimated based on the concentration of MC during the process. In addition, cell lysis was determined based on the concentration of dissolved MC during the treatment processes. Differences in MC concentration between the WTP abstraction points, raw water and during the treatment process were tested using the t-test and one-way repeated-measures ANOVA and TukeyHSD post hoc. However, when necessary, non-parametric tests, i.e., Mann–Whitney, were applied.

The differences of MC concentrations among the sampled sites in MB and NG were tested using a one-way ANOVA. Later, the sum of the identified MC structural variants was summarized as MC-LR equivalents in order to explore the relationship with environmental variables. We predicted the MC concentrations using a support vector classifier (SVC) using the SVM function in caret package in R for four categories of MC concentration: low (<1 µg MC-LR equiv. L^−1^), moderate (1–5 µg MC-LR equiv. L^−1^), high (6–10 µg MC-LR equiv. L^−1^) and very high (>10 µg MC-LR equiv. L^−1^). The data used for the SVC were centered and scaled to reduce the variability in the datasets.

A partial least square (PLS) regression model was used to explore the dependence of MC concentrations on environmental factors, including the cell abundance or biovolume of the two toxigenic cyanobacteria, *Microcystis* spp. and *Dolichospermum* sp., and other environmental factors that can cause variability in MC production, such as temperature, light, water column stability, pH, nutrients (NO3^−^, NH4^+^, SRSi, SRP and N:P ratio) and meteorological variables (mean air temperature, mean wind speed for the five days (D5), total rainfall for the five days (D5) and mean solar radiation for the ten days (D10) prior to sampling) and wind direction on the day of sampling. Correlations between the variables are shown in Table S5. Data in NG and MB were pooled together, and the Pearson product–moment partial correlation coefficient was used to explore the correlations between MC concentrations and exploratory variables from the PLS model. All statistical tests and graphical outputs were produced using R (version 4.0.2) integrated in RStudio environment (version 1.3.1093).

## Figures and Tables

**Table 1:** Standardized parameter coefficients from the global partial least square (PLS) regression models using log_10_ transformed variables to predict MC concentration in NG and MB, as well as correlation coefficients between MC concentration and environmental and biological parameters. Data from NG and MB were pooled together, and the Pearson product–moment partial correlation coefficient was used. Significant variables are marked in bold, statistically significant coefficients are indicated by asterisks: *** *p* < 0.001; ** p < 0.01; * *p* < 0.05.

Parameters	Min–Max (Mean ± SD)	Regression Coefficient (Log_10_)	Correlation Coefficient
Intercept		−2.044	
SRP (µg L^−1^)	0.56–380.3 (13.4 ± 38.8)	0.055	0.31
NH_4_ (µg L^−1^)	0.01–1206.8 (49.3 ± 150.9)	0.041	0.32
NO_3_^−^ (µg L^−1^) ^#^	1.11–947.4 (73.5 ± 127)	-	0.02
NO_2_^−^ (µg L^−1^) ^#^	0.00–47.2 (4.7 ± 7.1)	-	0.24
N:P ratio	0.16–149.1 (18.2 ± 22.3)	-0.093	0.02
Turbidity (NTU)	0.02–197.2 (18.2 ± 22.3)	-	0.42
Rainfall (mm)	0–61.1 (11.2 ± 13.9)	0.004	0.06
Wind speed (km h^−1^)	3.1–8.1 (4.5 ± 1.1)	−0.703 *	0.31
Wind direction	N-S, E-W, S-N, N-NE (S-SE, E-W)	0.035 *	0.18
Solar radiation (Wm^2^ day^−1^)	181.7–394.7 (290.9 ± 80.9)	1.021 ***	0.60
*Microcystis* biovolume (mm^3^ L^−1^)	0.27 -69 (7.3 ± 9.4)	-	0.62
*Microcystis* cell number	39,000–1,061,206 (106,277 ± 148,826)	0.031 **	0.57
*Dolichospermum* biovolume (mm^3^ L^−1^)	0.07–20.6 (3.9 ± 4.1)	-	0.22
*Dolichospermum* cell number	645–182,627 (33,822 ± 34,975)	0.008	0.24
R^2^		0.49	

^#^ Dissolved fractions of N used for the calculation of the N:P ratio applied in the PLS model.

**Table 2:** Main characteristics of the two water treatment plants (Gaba III and Walukuba) and physical-chemical characteristics of water (mean ± SD) collected during the water treatment processes between November 2016 and January 2017. MB: Murchison Bay, NG: Napoleon Gulf (*n* = 90, 45 samples for each WTP). Note: Treated water production as inferred from National Water and Sewerage Corporation (NWSC) annual report (2020). The number of consumers is based on the Uganda National Household Survey (2019/2020) assuming an average of 4.5 persons per household.

Capacity (m^3^.day^−1^)	Piped Water Connection	Demand (m^3^/day)	Treatment Steps		Physical-Chemical Parameters during the Treatment Process				
					Depth-Integrated Lake Water (at WTP Abstraction Point)	Raw Water (Entry Point of the WTP)	After Flocculation	After Sand Filtration	Finished Water
MB-Gaba									
80,000, but supplementing Gaba I and II water treatment plants	315,897 connections supplying between 1,421,536 and 2 million people in Kampala (including lake shore residents)	300,000	Pre-chlorination, coagulation, clarification, rapid sand filtration, pH correction and chlorination	pH	8.0 ± 0.3	7.6 ± 0.1	7.2 ± 0.3	7.2 ± 0.3	7.0 ± 0.2
				Conductivity (μS cm^−1^)	118 ± 2.1	119 ± 3.7	129 ± 5.5	129 ± 5.9	131 ± 5.3
				Temperature (°C)	26.1 ± 0.3	26.1 ± 0.4	26.0 ± 0.4	26.0 ± 0.3	26.4 ± 0.4
				Dissolved oxygen (mg L^−1^)	5.0 ± 0.7	5.9 ± 0.4	7.1 ± 0.1	7.4 ± 0.1	7.1 ± 0.1
				Dissolved oxygen (%)	71.0 ± 9.6	85.0 ± 8.3	102 ± 1.7	104 ± 1.2	101 ± 1.2
NG-Walukuba									
50,000, but producing only 30,100	28,881 connections supplying between 129,964 and 500,000 people (including the neighboring districts of Iganga, Buikwe and Mayuge)	30,000	Coagulation, clarification, rapid sand filtration and chlorination	pH	8.9 ± 0.3	7.8 ± 0.2	7.5 ± 0.4	7.4 ± 0.2	7.6 ± 0.2
				Conductivity (μS cm^−1^)	102 ± 1.2	104 ± 6.7	105 ± 6.8	103 ± 2.8	109 ± 4.3
				Temperature (°C)	27.1 ± 0.3	27.3 ± 0.6	27.3 ± 0.4	27.0 ± 0.4	27.2 ± 0.2
				Dissolved oxygen (mg L^−1^)	6.6 ± 1.0	3.8 ± 0.9	3.9 ± 0.6	3.2 ± 0.8	6.4 ± 0.6
				Dissolved oxygen (%)	94.3 ± 15.1	55.6 ± 13	56.7 ± 9.5	47 ± 11.8	92.1 ± 8.9

## Data Availability

The data presented in this study are available in Supplementary Material (Figures S1–S5; Tables S1–S5). The field survey data presented in this study are available on request from the corresponding author (C.B.). The WTP survey data presented in this study are available on request from the corresponding author (R.K.).

## Supplementary Materials

The following supporting information can be downloaded at: https://www.mdpi.com/article/10.3390/toxins14100664/s1, Figure S1: The temporal dynamics of the most abundant toxigenic cyanobacteria (see Figure 1) recorded from the lake survey at the sampling stations: (i) inshore, (ii) recreational area, (iii) WTP abstraction point (Abs. point), (iv) open lake, in Murchison Bay and Napoleon Gulf. (Data collected between November 2017 and October 2018, *n* = 120); Figure S2: The mean (±SD) MC concentration (A), total cyanobacteria biovolume (B) and toxigenic cyanobacteria biovolume (C) at the WTP abstraction point (Abs. point) in Murchison Bay and Napoleon Gulf during WTP survey 1 (November 2016–January 2017) and lake survey 2 (November 2017–October 2018); Figure S3: The temporal variation in intracellular MC concentration (MC-LR equiv.) recorded from Murchison Bay (upper part) and Napoleon Gulf (lower part). Note: Abs. point = WTP abstraction point (Data collected between November 2017 and October 2018, *n* = 96). MC-YR was detected only once (0.29 µg MC-LR equiv. L^−1^ in June 2018) at inshore station of Murchison Bay. Note that the scales differ between MB and NG; Figure S4: Recovery of MC-LR dissolved in GF/C filtered lake water (Mondsee), treated water and Millipore water using C18 SPE columns (Sep-Pak^®^ Vac tC18 cartridge 100 mg) which have been either eluted immediately, or after drying at 50 °C and stored frozen (−20 °C) (24 h). The UV chromatogram area (A), and extracted ion chromatogram (EIC) areas (B) from LC-MS analysis are shown; Figure S5: LC-MS chromatograms (UV absorption at 240 nm) for particulate and dissolved MC structural variants recorded from water samples and during treatment steps including abstraction, raw water, flocculation, sand filtration, and finished water. For clarity MS base peak spectra were omitted. Elution of indicated MC structural variants was confirmed through ion extraction, i.e. MC-RR [M+H]^+^ 1038.5, MeSerMC-YR [M+H]^+^ 1063.5, MC-YR [M+H]^+^ 1045.5, MC-LR [M+H]^+^ 995.5, DM-MC-RY [M+H]^+^ 1031.5, MC-RY [M+H]^+^ 1045.5. (**A**) Murchison Bay (Gaba III WTP), from 22 November 2016, (**B**) Napoleon Gulf (Walukuba WTP), from 20 December 2016, (**C**) Chromatographic and fragmentation patterns of the common MC analogues MC-LR, -RR and -YR recorded during the field survey (November 2017–October 2018); Table S1: The frequency of detection (percentage) of dissolved and intracellular MC detected and identified during the water treatment process in MB-Gaba and NG-Walukuba (*n* = 90, with 45 samples from MB-Gaba and NG-Walukuba each, i.e., 9 samples from each treatment step). Abbreviations: Abs. point = depth-integrated lake water at WTP abstraction point, RW = Raw water (entering water treatment), CFW = Coagulated and flocculated water, SFW = Sand filtered water and FW = Final water and (-) = not detected; Table S2: Mean (±SD) dissolved MC in lake water in MB and NG, and during the treatment processes, as analyzed by ELISA (*n* = 90) and HPLC (*n* = 90). Abbreviations: Abs. point = depth-integrated lake water at WTP abstraction point, RW = Raw water (entering water treatment), CFW = Coagulated and flocculated water, SFW = Sand filtered water and FW = Final water; Table S3: Recovery of MC-LR, MC-RR, MC-YR added to GF/C filtered water (1.0 µg MC-LR equiv. L^−1^) to control for SPE conditions during individual water treatments. MB denotes Murchison Bay, and NG is Napoleon Gulf. The hyphen “-” means not spiked; Table S4: List of cyanobacteria strains used as controls for cyanotoxin extraction and detection via LC-MS/MS (during field survey, November 2017–October 2018). Strains used as a reference for cyanotoxins were cultured at Muséum National d’Histoire Naturelle (MNHN) (PMC = Paris Museum Collection) or at Research Department for Limnology in Mondsee (MU = Murchison Bay, Lake Victoria, 19G6 = Lake George and NAP = Napoleon Gulf, Lake Victoria). √, means detected; –, means not detected; Table S5: Pearson correlation coefficients calculated between environmental parameters recorded during field sampling (*n* = 120) between November 2017 and October 2018.
