# Supplementary material for: Characterization of Potential Threats from Cyanobacterial Toxins in Lake Victoria Embayments and during Water Treatment
Source: Toxins (Basel). 2022 Sep 23;14(10):664. doi: 10.3390/toxins14100664 (PMC9607203; doi:10.3390/toxins14100664)
Supplement: Supplementary file 1 [file toxins-14-00664-s001.zip › toxins-1853809-supplementary.pdf]

toxins

Supplementary Materials: Characterization of Potential Threats from Cyanobacterial Toxins in Lake Victoria Embayments and during Water Treatment

Mark Olokotum, Jean-François Humbert, Catherine Quiblier, William Okello, Ronald Semyalo, Marc Troussellier, Benjamin Marie, Kathrin Baumann, Rainer Kurmayer and Cécile Bernard

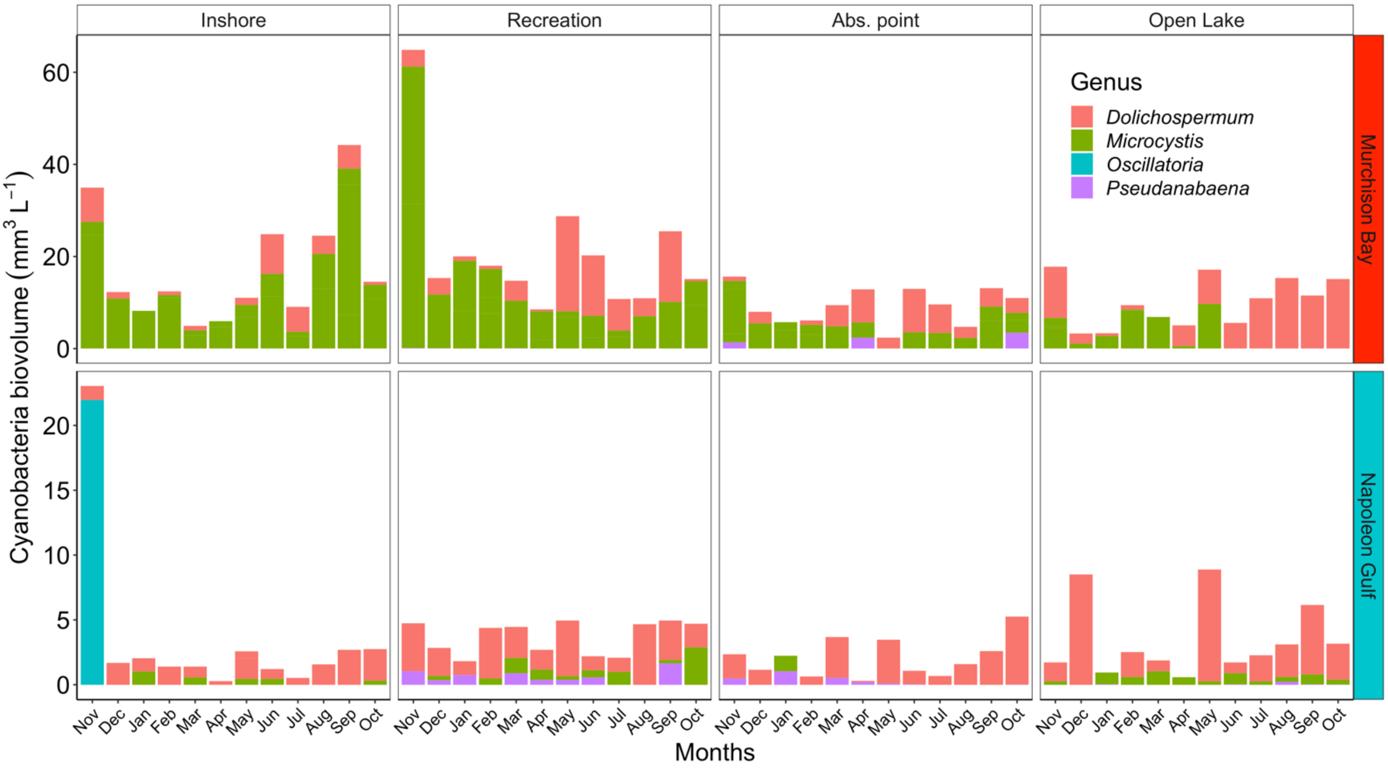

Figure S1. The temporal dynamics of the most abundant toxigenic cyanobacteria (see Figure 1) recorded from the lake survey at the sampling stations: (i) inshore, (ii) recreational area, (iii) WTP abstraction point (Abs. point), (iv) open lake, in Murchison Bay and Napoleon Gulf. (Data collected between November 2017–October 2018, $n = 120$).

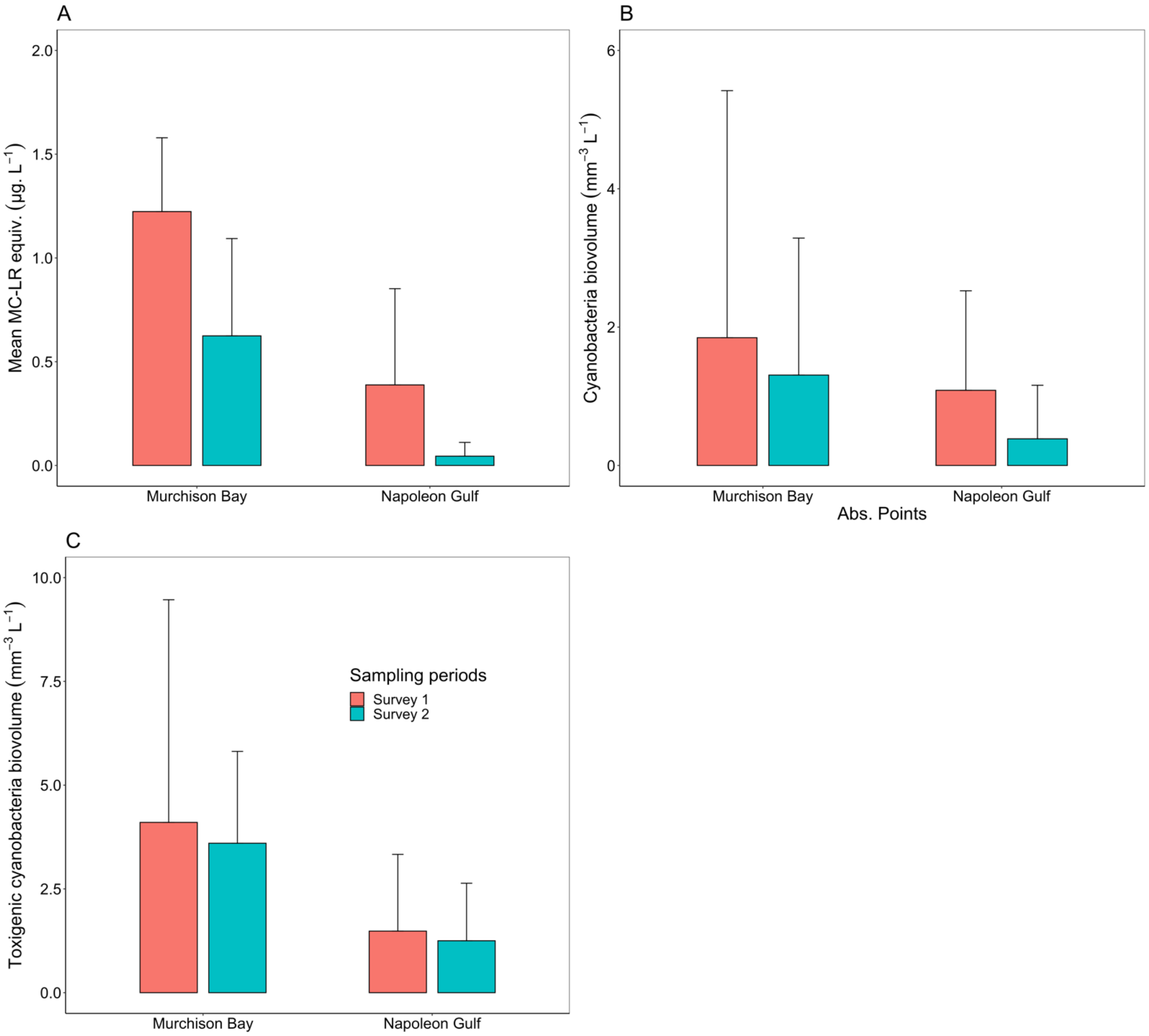

Figure S2. The mean (±SD) MC concentration (A), total cyanobacteria biovolume (B), and toxigenic cyanobacteria biovolume (C) at the WTP abstraction point (Abs. point) in Murchison Bay and Napoleon Gulf during WTP survey 1 (November 2016–January 2017) and lake survey 2 (November 2017–October 2018).

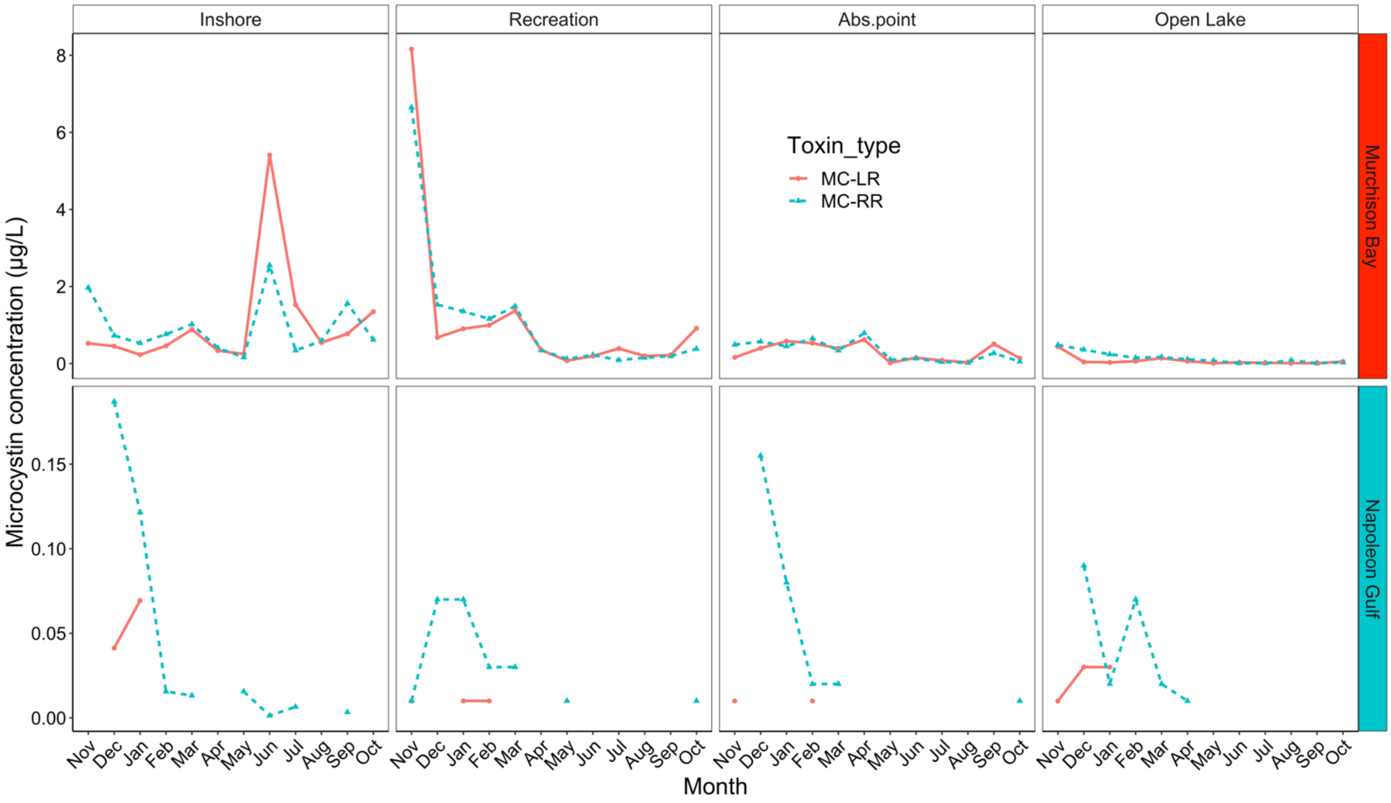

Figure S3. The temporal variation in intracellular MC concentration (MC-LR equiv.) recorded from Murchison Bay (upper part) and Napoleon Gulf (lower part). Note: Abs. point = WTP abstraction point (Data collected between November 2017–October 2018, n = 96). MC-YR was detected only once (0.29 µg MC-LR equiv. L^{-1} in June 2018) at inshore station of Murchison Bay. Note that the scales differ between MB and NG.

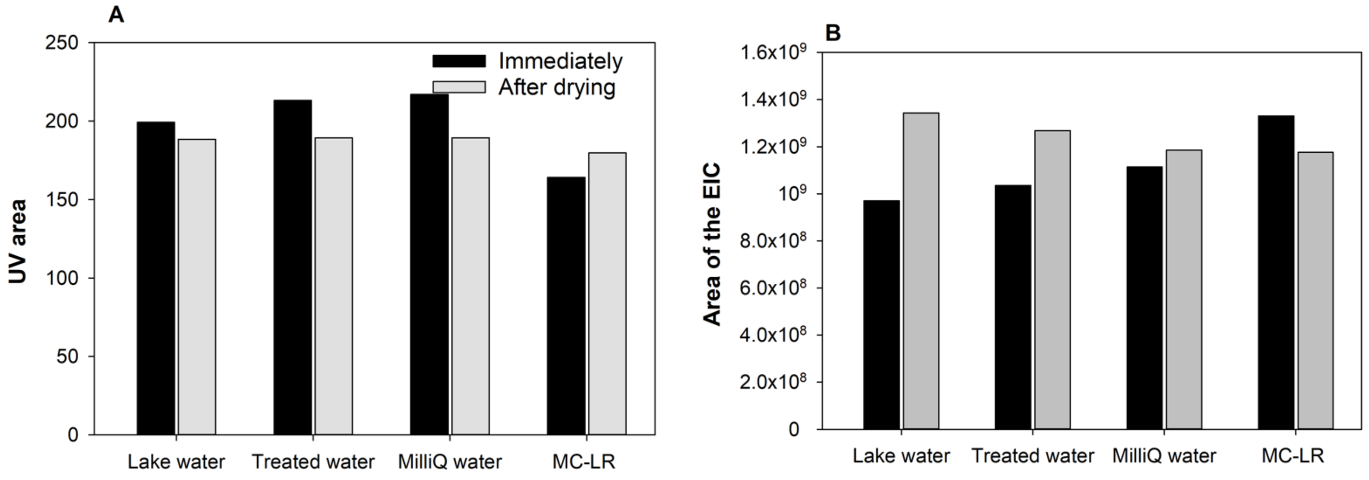

Figure S4. Recovery of MC-LR dissolved in GF/C filtered lake water (Mondsee), treated water and Millipore water using C18 SPE columns (Sep-Pak® Vac tC18 cartridge 100 mg) which have been either eluted immediately, or after drying at 50 °C and stored frozen (−20 °C) (24 h). The UV chromatogram area (A), and extracted ion chromatogram (EIC) areas (B) from LC-MS analysis are shown.

A) Murchison Bay (Gaba III WTP)

B) Napoleon Gulf (Walukuba WTP)

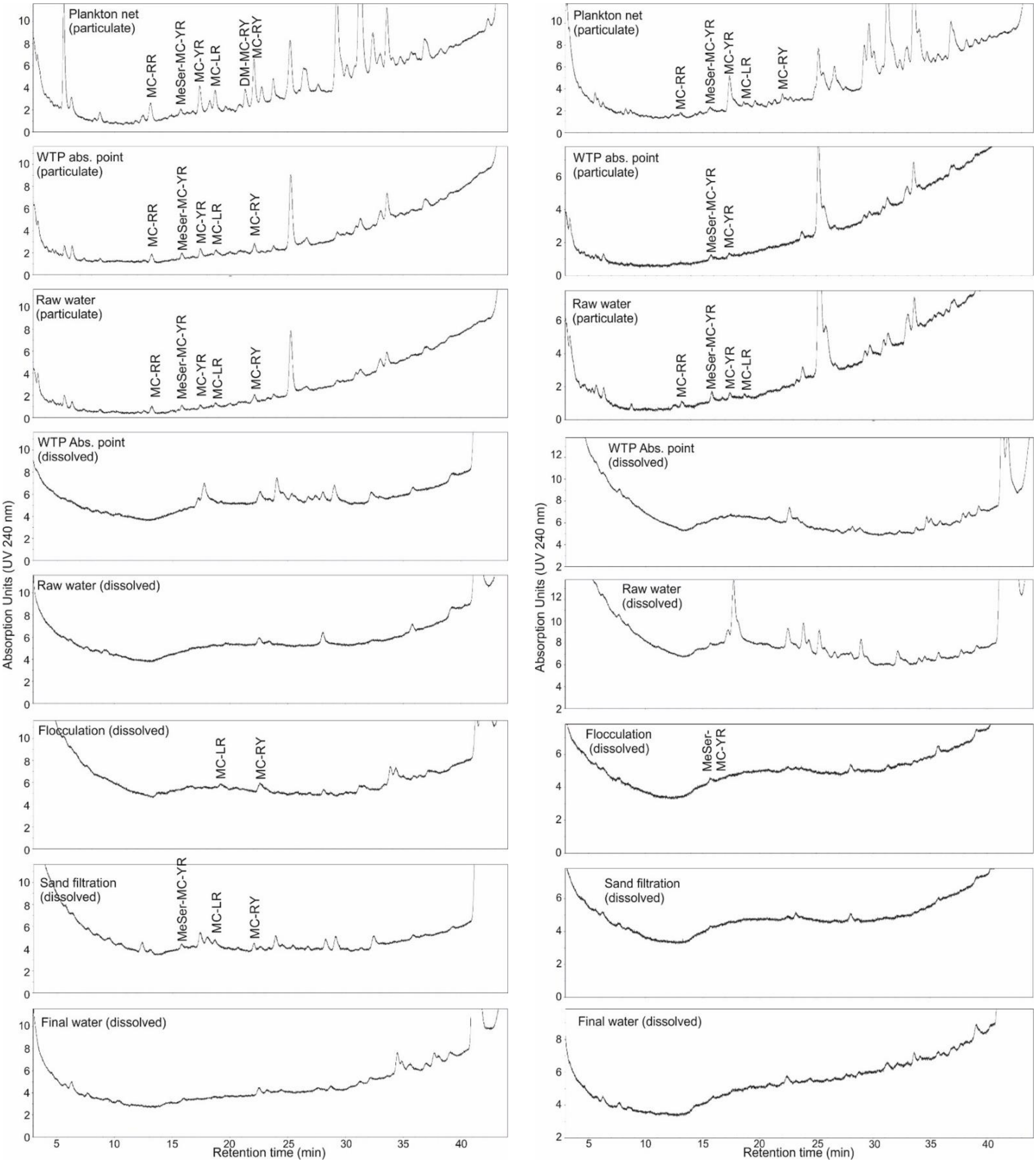

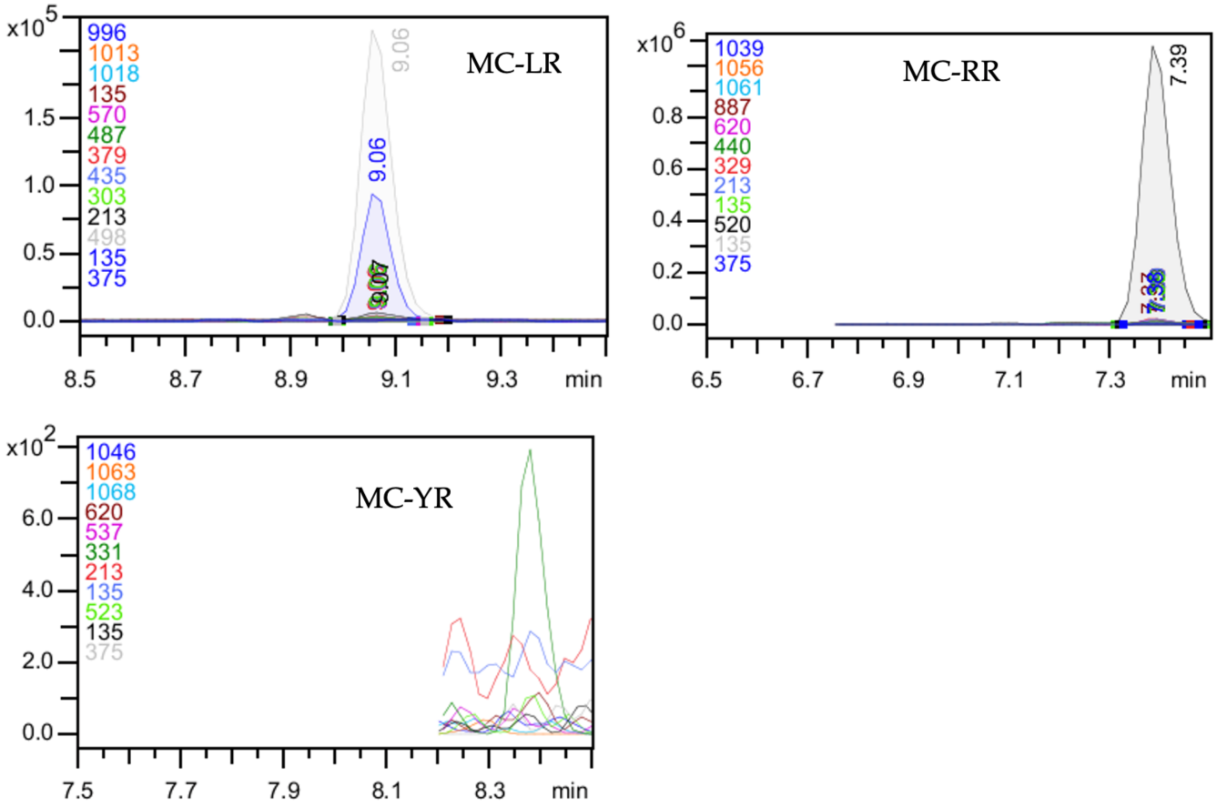

Figure S5. LC-MS chromatograms (UV absorption at 240 nm) for particulate and dissolved MC structural variants recorded from water samples and during treatment steps including abstraction, raw water, flocculation, sand filtration, and finished water. For clarity MS base peak spectra were omitted. Elution of indicated MC structural variants was confirmed through ion extraction, i.e. MC-RR $[M+H]^+$ 1038.5, MeSerMC-YR $[M+H]^+$ 1063.5, MC-YR $[M+H]^+$ 1045.5, MC-LR $[M+H]^+$ 995.5, DM-MC-RY $[M+H]^+$ 1031.5, MC-RY $[M+H]^+$ 1045.5. (**A**) Murchison Bay (Gaba III WTP), from 22 November 2016, (**B**) Napoleon Gulf (Walukuba WTP), from 20 December 2016, (**C**) Chromatographic and fragmentation patterns of the common MC analogues MC-LR, -RR and -YR recorded during the field survey (November 2017–October 2018).

Table S1. The frequency of detection (percentage) of dissolved and intracellular MC detected and identified along the water treatment process in MB-Gaba and NG-Walukuba (*n* = 90, with 45 samples from MB-Gaba and NG-Walukuba each, i.e., 9 samples from each treatment step). Abbreviations: Abs. point = depth-integrated lake water at WTP abstraction point, RW = Raw water (entering water treatment), CFW = Coagulated and flocculated water, SFW = Sand filtered water, and FW = Final water and (-) = not detected.

**Dissolved MC**	**Ret. time (min)**	**Mass [M+H]⁺**	**MB-Gaba**					**NG-Walukuba**				
			**Abs. point**	**RW**	**CFW**	**SFW**	**FW**	**Abs. point**	**RW**	**CFW**	**SFW**	**FW**
MC-RR	13.1-13.3	1038.5	-	-	55.6	77.8	-	-	-	-	-	-
[NMeSer^{7}]-MC-YR	15.7-16.1	1063.5	-	-	44.4	88.9	11.1				-	-
MC-YR	17.3-17.5	1045.5	44.4	-	44.4	66.7	-	-	-	-	-	-
MC-LR	18.6-18.9	995.5	-	-	55.6	100	-	-	-	-	-	-
[Asp^{3}]-MC-RY	20.8	1031.5	-	-	-	11.1	-	-	-	-		-
[MeAsp^{3}]-MC-RY	22.1-22-2	1045.5	11.1	22.2	77.8	77.8	-		-	-	-	
**Intracellular MC**			**MB-Gaba**					**NG-Walukuba**				
			**Abs. point**	**RW**	**CFW**	**SFW**	**FW**	**Abs. point**	**RW**	**CFW**	**SFW**	**FW**
MC-RR	13.1-13.3	1038.5	100	33.3	-	-	-	44.4	-	-	-	-
[NMeSer^{7}]-MC-YR	15.7-16.1	1063.5	88.9	33.3	-	-	-	88.9	-	-	-	-
MC-YR	17.3-17.5	1045.5	100	33.3	-	-	-	66.7	-	-	-	-
MC-LR	18.6-18.9	995.5	100	33.3	-	-	-	44.4	-	-	-	-
[Asp^{3}]-MC-RY	20.8	1031.5	55.6	22.2	-	-	-	-	-	-	-	-
[MeAsp^{3}]-MC-RY	22.1-22-2	1045.5	100	33.3	-	-	-	-	-	-	-	-

Table S2. Mean (±SD) dissolved MC in lake water in MB and NG, and during the treatment processes as analysed by ELISA (*n* = 90) and HPLC (*n* = 90). Abbreviations: Abs. point = depth-integrated lake water at WTP abstraction point, RW = Raw water (entering water treatment), CFW = Coagulated and flocculated water, SFW = Sand filtered water, and FW = Final water.

**Station/Site**	**Treatment**	**MC (μg/L) (mean ± SD)**	
		ELISA	HPLC-MS
MB-Gaba	Abs. point	0.26 ± 0.08	0.85 ± 0.53
	RW	0.46 ± 0.17	0.13 ± 0.12
	CFW	0.53 ± 0.41	0.33 ± 0.36
	SFW	1.05 ± 0.75	0.10 ± 0.05
	FW	0.02 ± 0.001	0.02 ± 0.05
NG-Walukuba	Abs. point	0.13 ± 0.02	
	RW	0.12 ± 0.07	
	CFW	0.03 ± 0.02	
	SFW	0.04 ± 0.03	
	FW	0.04 ± 0.00	

Table S3. Recovery of MC-LR, MC-RR, MC-YR added to GF/C filtered water (1.0 µg MC-LR equiv. L^{-1}) to control for SPE conditions during individual water treatments. MB denotes Murchison Bay and NG is Napoleon Gulf. The hyphen "-" means not spiked.

	MB-Gaba (µg/L)			NG-Walukuba (µg/L)		
Water sample	MC-RR	MC-YR	MC-LR	MC-RR	MC-YR	MC-LR
Depth-integrated lake water (Abs. point)	1.60	1.63	1.45	1.43	1.75	1.91
Raw water	-	2.09	-	1.49	-	1.04
Coagulated and flocculated water	1.03	1.31	1.35	-	-	-
Sand filtered water	-	-	-	1.49	1.63	1.78
Final water	-	-	-	-	-	1.29

Table S4. List of cyanobacteria strains used as controls for cyanotoxin extraction and detection via LC-MS/MS (during field survey November 2017–October 2018). Strains used as a reference for cyanotoxins were cultured at Muséum National d'Histoire Naturelle (MNHN) (PMC = Paris Museum Collection) or at Research Department for Limnology in Mondsee (MU = Murchison Bay, Lake Victoria, 19G6 = Lake George and NAP = Napoleon Gulf, Lake Victoria). √, means detected; –, means not detected.

**Strain No.**	**Culture collection**	**Anatoxin-a**	**Homoanatoxin**	**Cylindrospermopsin**	**Saxitoxins**	**MC-LR, RR, YR**
parent ion [M+H]+		166.1226	173.1310	416.1234	300.1415	995.56/1038.57/1045.54
Fragmentation products [M+H]+		149.097/ 131.086	163.12/ 145.10/135.12/ 162.13	176.12/194.13/224.13/ 274.09/301.13/318.16	282.13/179.09/ 258.12/186.10	135.08/213.08/375.19
Retention time (min)		1.2	1.8	1.7	0.7	8.8/7.3/8.7
Phormidium favosum (PMC 972.16)	MNHN	√	–	–	√	–
Phormidium favosum (PMC 974.16,)	MNHN	–	√	–	–	–
Phormidium favosum (PMC 240.04)	MNHN	√	√		–	–
Raphidiopsis raciborskii (PMC 99.03),	MNHN	–	–	√	–	–
Aphanizomenon gracile (PMC 638.10)	MNHN	–	–	–	√	
Microcystis aeruginosa (PMC 728.11);	MNHN	–	–	–	–	√
Microcystis sp. PMC 1082.18	MNHN	–	–	–	–	–
Microcystis sp. PMC 1083.18	MNHN	–	–	–	–	–
Microcystis sp. PMC 1084.18	MNHN	–	–	–	–	–
Microcystis sp. MU09	MNHN	–	–	–	–	–
Microcystis sp. MU17	Mondsee	–	–	–	–	–
Microcystis sp. 19G6	Mondsee	–	–	–	–	–
Microcystis sp. NAP07	Mondsee	–	–	–	–	√
Microcystis sp. NAP17	Mondsee	–	–	–	–	

Table S5. Pearson correlation coefficients calculated between environmental parameters recorded during field sampling (n = 120) between November 2017–October 2018.

	Secchi	Sampling Depth	Temp	pH	Oxygen (mg/L)	Oxygen (%)	Turbidity	SRP	NO3	NH4	N:P	SRSi	TN	TP	Rainfall_D5	MeanSolar Radiation_D10	MeanAir Temp	MeanRelativeHumidity	MeanWind speed_D5
Secchi (m)																			
Sampling depth (m)	0.67																		
Temperature (°C)	0.09	-0.03																	
pH	0.22	0.13	0.47																
Oxygen (mg/L)	0.16	0.01	0.27	0.64															
Oxygen saturation (%)	0.15	0.00	0.33	0.65	1.00														
Turbidity (NTU)	-0.36	-0.36	0.04	-0.08	0.10	0.11													
SRP (μg/L)	-0.06	-0.02	0.14	-0.04	0.02	0.04	0.59												
NO3 (μg/L)	0.11	0.14	0.00	-0.10	0.07	0.07	0.18	0.34											
NH4 (μg/L)	-0.17	-0.11	0.08	-0.03	-0.08	-0.07	0.38	0.58	0.29										
N:P	0.03	0.13	0.03	-0.06	-0.01	-0.01	0.19	0.23	0.82	0.38									
SRSi (μg/L)	-0.21	-0.11	0.24	0.00	0.14	0.15	0.47	0.68	0.31	0.49	0.20								
TN (μg/L)	-0.12	-0.05	0.23	-0.02	0.11	0.13	0.52	0.39	0.27	0.48	0.39	0.34							
TP (μg/L)	-0.14	-0.04	0.30	0.15	0.31	0.34	0.46	0.44	0.28	0.45	0.26	0.51	0.71						
Rainfall_D5 (mm)	0.15	0.10	0.08	-0.05	0.06	0.06	0.10	0.16	0.55	0.10	0.43	0.10	0.16	0.13					
MeanSolarRadiation_D10 (W/m2)	-0.67	-0.26	-0.26	-0.17	-0.06	-0.07	0.38	0.21	0.10	0.31	0.23	0.25	0.25	0.27	-0.09				
MeanAirTemp (°C)	-0.34	-0.07	0.23	0.12	0.11	0.13	0.33	0.30	0.01	0.19	0.05	0.25	0.32	0.34	-0.17	0.59			
MeanRelativeHumidity (%)	0.21	0.08	-0.13	-0.28	-0.03	-0.05	0.06	0.10	0.19	0.12	0.19	0.00	0.08	0.06	0.10	-0.10	-0.50		
MeanWindspeed_D5 (km/s)	-0.25	-0.11	-0.46	-0.07	0.24	0.21	0.29	0.25	0.14	0.32	0.17	0.20	0.17	0.22	-0.13	0.65	0.28	0.10	
Wind direction from (°)	-0.07	0.03	0.04	0.15	0.19	0.19	0.12	0.10	0.26	0.15	0.22	0.27	0.10	0.18	0.20	0.12	0.14	-0.42	0.24
